# Supplementary material for: Mapping Neutralizing Antibody Epitope Specificities to an HIV Env Trimer in Immunized and in Infected Rhesus Macaques
Source: Cell Rep. 2020 Sep 8;32(10):108122. doi: 10.1016/j.celrep.2020.108122 (PMC7487785; doi:10.1016/j.celrep.2020.108122)
Supplement: Document S1. Figures S1–S5 and Tables S1–S4 [file mmc1.pdf]

Cell Reports, Volume *32*

Supplemental Information

Mapping Neutralizing Antibody Epitope Specificities to an HIV Env Trimer in Immunized and in Infected Rhesus Macaques

Fangzhu Zhao, Collin Joyce, Alison Burns, Bartek Nogal, Christopher A. Cottrell, Alejandra Ramos, Trevor Biddle, Matthias Pauthner, Rebecca Nedellec, Huma Qureshi, Rosemarie Mason, Elise Landais, Bryan Briney, Andrew B. Ward, Dennis R. Burton, and Devin Sok

Supplemental Information

Mapping neutralizing antibody epitope specificities to an HIV Env trimer in immunized and in infected rhesus macaques

Fangzhu Zhao, Collin Joyce, Alison Burns, Bartek Nogal, Christopher A. Cottrell, Alejandra Ramos, Trevor Biddle, Matthias Pauthner, Rebecca Nedellec, Huma Qureshi, Rosemarie Mason, Elise Landais, Bryan Briney, Andrew B. Ward, Dennis R. Burton, Devin Sok

A

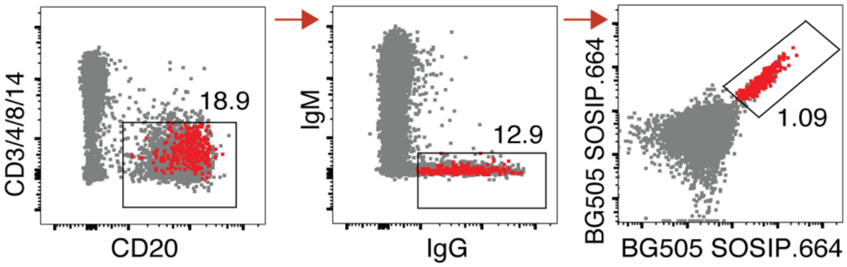

B

Group	Sample	Sorted Cells	IgG⁺ Wells	Neut⁺ Wells	Cloned mAbs
IMM	12-084_WK25	524	188	3	3
IMM	12-046_WK26	723	155	4	2
IMM	4O9_WK68	562	80	9	5
IMM	12-137_WK26	768	224	0	0
IMM	12-143_WK26	418	136	0	0
NON-INF	4O9_WK88	76	17	0	0
NI	BZ31_WK86	240	81	5	3
NI	BZ05_WK86	220	32	4	3
IMM	11M088_WK28	720	139	2	1
IMM	12M169_WK28	240	43	0	0
NI	BZ11_WK86	1008	378	1	1
NI	BZ15_WK86	1056	331	0	0
NON-INF	4O9_WK76	484	158	4	3
NON-INF	12-046_WK76	198	44	6	4
INF	11M088_WK86	440	73	11	10
INF	12M169_WK86	176	31	4	3
IMM	12-143_WK26	792	272	1	1
IMM	12M169_WK26	528	159	2	1
INF	12-143_WK70	396	163	1	1
IMM	12-137_WK26	1056	393	5	4
IMM	12-137_WK76	264	98	2	2
	**Total**	**10889**	**3195**	**64**	**47**

Figure S1. FACS sorting and neutralization screening details. Related to Figure 1. (**A**) FACS sorting layout from rhesus PBMCs using biotinylated BG505 SOSIP.664-Avi after gating on lymphocytes/singlets. Events that are BG505 SOSIP.664^{++} are shown in red. (**B**) Summary number of sorted cells, wells with detectable secreted IgG, wells with positive BG505 neutralization, and cloned mAbs from all rhesus PBMCs samples.

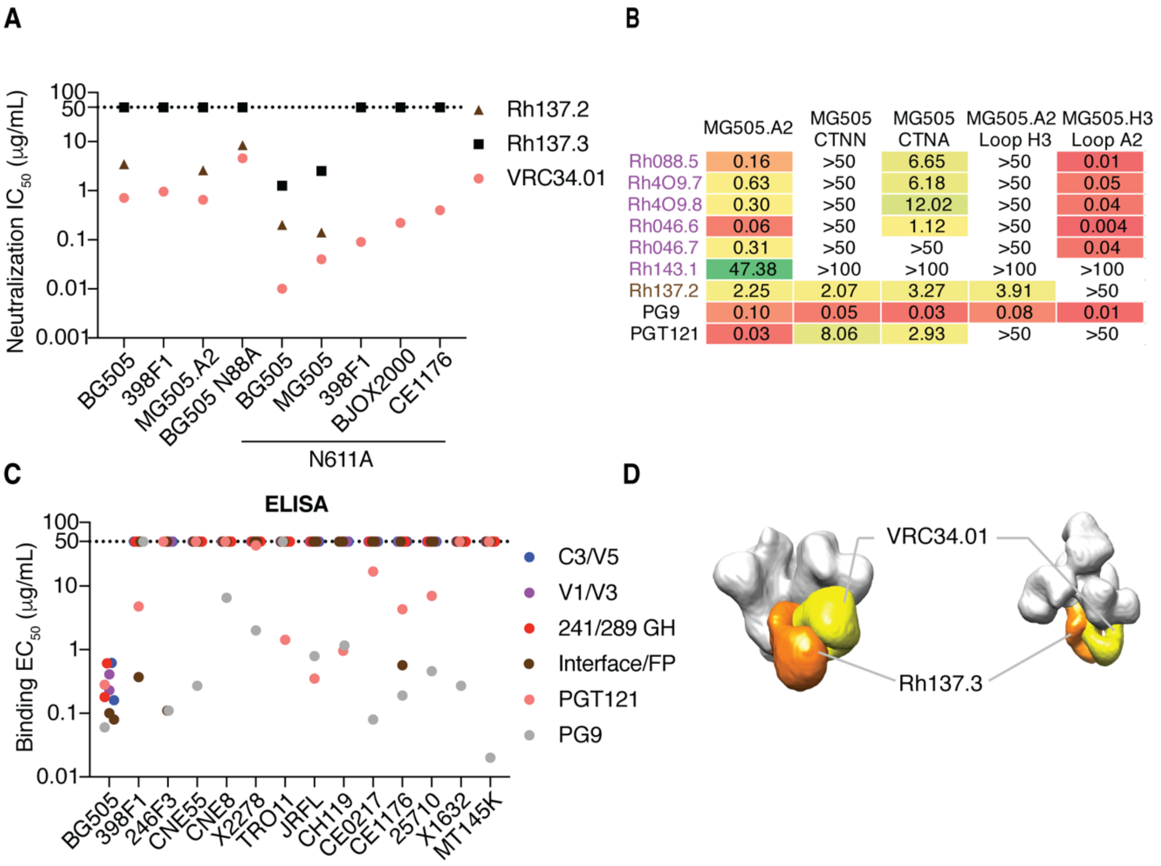

	MG505.A2	MG505 CTNN	MG505 CTNA	MG505.A2 Loop H3	MG505.H3 Loop A2
Rh088.5	0.16	>50	6.65	>50	0.01
Rh4O9.7	0.63	>50	6.18	>50	0.05
Rh4O9.8	0.30	>50	12.02	>50	0.04
Rh046.6	0.06	>50	1.12	>50	0.004
Rh046.7	0.31	>50	>50	>50	0.04
Rh143.1	47.38	>100	>100	>100	>100
Rh137.2	2.25	2.07	3.27	3.91	>50
PG9	0.10	0.05	0.03	0.08	0.01
PGT121	0.03	8.06	2.93	>50	>50

Figure S2. BG505 nAbs remain autologous and do not bind other HIV Env trimers. Related to Figure 2. (**A**) Neutralization IC_{50}s evaluated for gp120-gp41 interface-targeting mAbs Rh137.2, Rh137.3, and including VRC34.01 as control bnAb. Antibodies were tested for neutralization against wildtype BG505, 398F1, MG505.A2 viruses, BG505 N88A mutant, and N611 glycan-deleted virus mutants. (**B**) Neutralization IC_{50}s evaluated for V1/V3-targeting mAbs (purple), gp120-gp41 interface-targeting mAb Rh137.3 (brown) and PGT121 and PG9 as control bnAbs. Antibodies were tested for neutralization against wildtype MG505.A2, MG505.A2 V1 loop mutants including CTNN, CTNA, and CTNNVTNA (Loop H3), and MG505.H3 V1 loop mutant (CTNVTNN). (**C**) ELISA binding EC_{50}s evaluated for two mAbs for each epitope specificity and including PGT121 and PG9 as controls. Antibodies were tested for binding against a set of HIV Env trimers from an indicator panel of global viruses and MT145K as a SIV Env trimer (Andrabi et al., 2019). (**D**) Overlap of fusion peptide epitopes targeted by VRC34.01 (yellow) and Rh137.3 (orange).

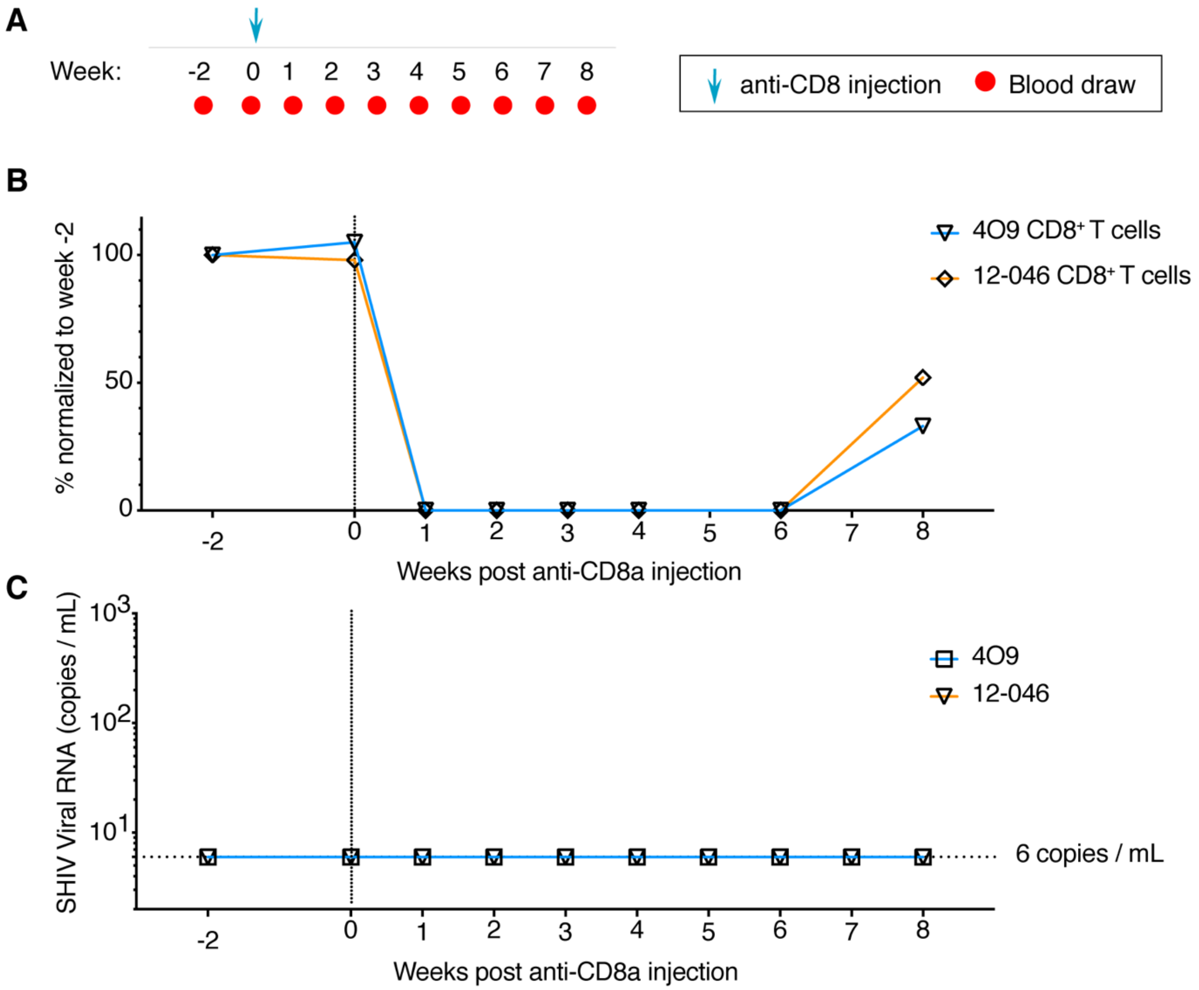

Figure S3. CD8+ T cell depletion does not affect protection against SHIV$_{BG505}$ challenge. Related to Figure 3. (**A**) Animals 12-046 and 4O9 were injected with anti-CD8a antibody after the SHIV challenge study. (**B**) CD8+ T cell frequency after anti-CD8a mAb injection normalized to week -2 in animals 12-046 (orange) and 4O9 (blue). (**C**) SHIV viral load in animals 12-046 (orange) and 4O9 (blue) after anti-CD8a antibody injection.

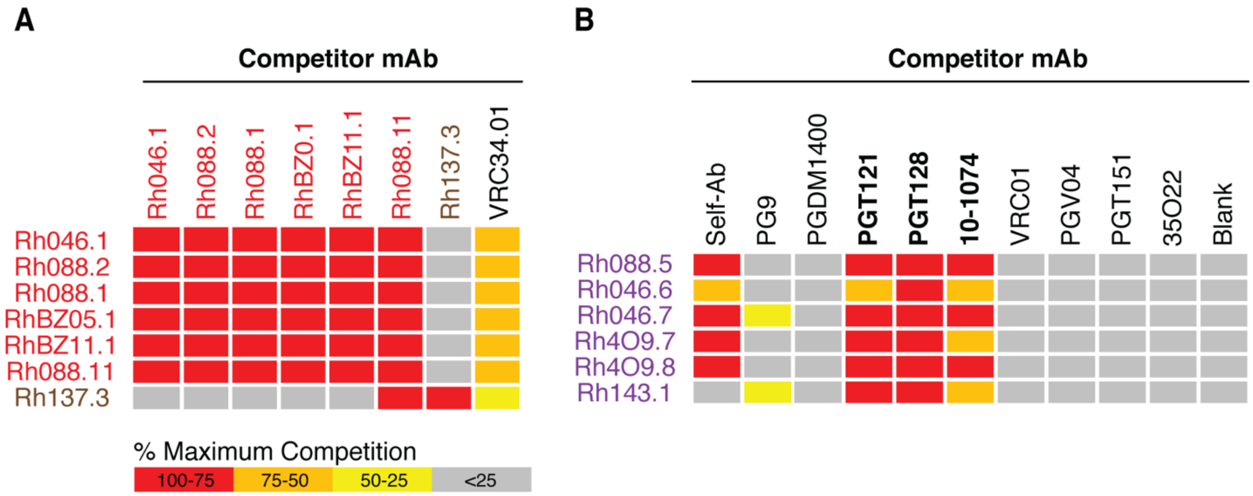

Figure S4. Competition ELISA of strain-specific nAbs and human bnAbs. Related to Figure 4. (A-B) ELISA competition between HIV-1 bnAbs and rhesus mAbs for binding to BG505 SOSIP and colored according to the key. (**A**) Competition between 241/289 GH-targeting mAbs, FP-targeting rhesus mAb Rh137.3, and human bnAb VRC34.01. (**B**) Competition between V1/V3-targeting mAbs and human HIV bnAbs. "Self-Ab" indicates competition between the labelled and unlabeled versions of the same rhesus mAb.

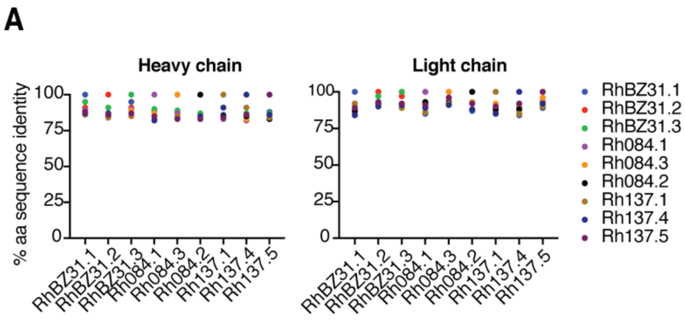

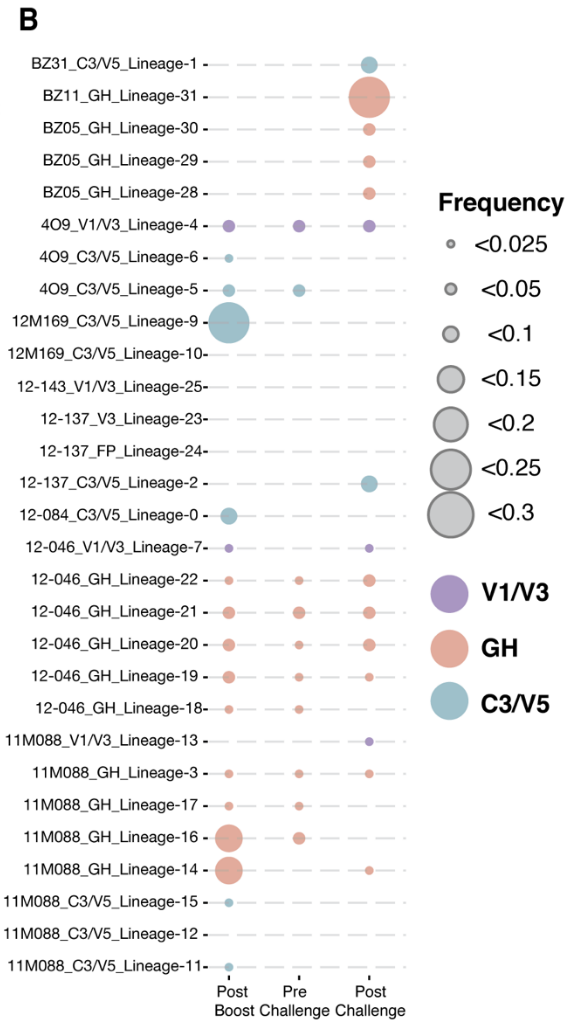

C

Replicate #1

	Post Boost	Pre-Challenge	Post Challenge
BZ31	N/A	N/A	465
12-084	0	N/A	N/A
12-137	0	0	26

Replicate #2

	Post Boost	Pre-Challenge	Post Challenge
BZ31	N/A	N/A	3056
12-084	176	N/A	N/A
12-137	0	0	55

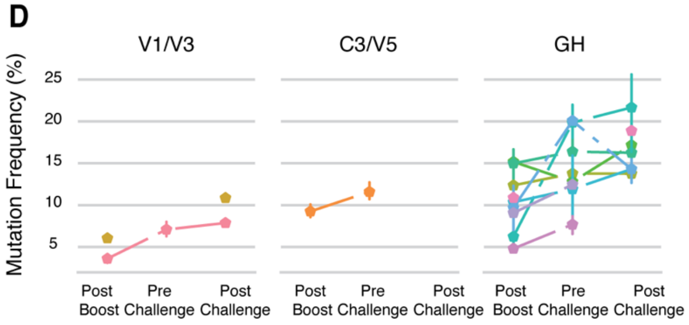

Figure S5. NAb frequency and mutation rate at multiple timepoints. Related to Figure 5. (**A**) Heavy chain and light chain amino acid sequence identities for rhesus mAbs. (**B**) nAb lineage heavy chain frequency (fractional abundance, defined as the number of unique antibody sequences in lineage divided by number of unique sequences in the repertoire at that timepoint) from corresponding animal NGS sequences at multiple timepoints: post-boost, pre-challenge, and post-challenge. (**C**) public C3/V5 heavy chain sequences found in animals BZ31, 12-084, 12-137 at multiple timepoints. Each replicate represents for an independent NGS run. N/A: not applicable. (**D**) Somatic hypermutation frequency of nAb lineages traced in (**B**) that can be found in more than one time-point. Each color represents for a separate lineage.

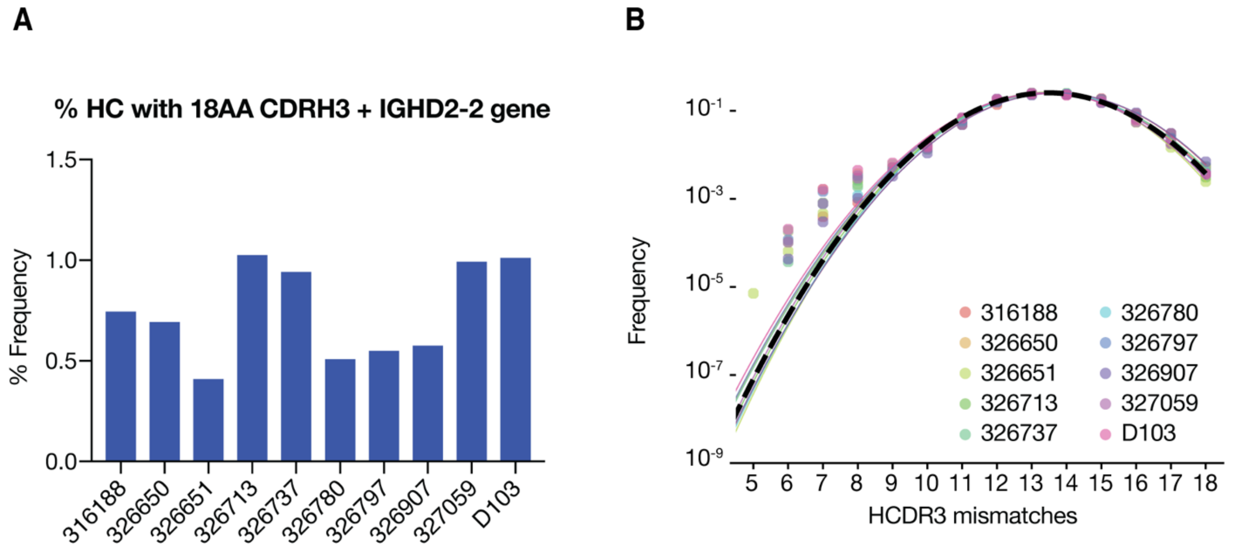

Figure S6. Frequency of similar public clonotype antibodies in the human B cell repertoire. Related to Figure 5. (**A**) Frequency of human heavy chain antibodies with an 18 amino acid CDRH3 length and containing the IGHD2-2 gene among ten healthy donors. (**B**) Distribution frequency of human heavy chain antibodies among 10 donors based on number of differences in amino acids compared to the most closely related NHP public clonotype antibody sequence.

Table S1. ELISA binding EC_{50} table of all rhesus mAbs against BG505 SOSIP, 398F1 SOSIP, and BG505 gp120. Related to Figure 1.

Group	Animal ID	mAb ID	Epitope	BG505 SOSIP EC_{50} (µg/mL)	BG505 gp120 EC_{50} (µg/mL)	398F1 SOSIP EC_{50} (µg/mL)
IMM	12-084	Rh084.1	C3/V5	0.62	0.22	>50
		Rh084.2	C3/V5	0.41	0.09	>50
		Rh084.3	C3/V5	0.66	0.17	>50
	4O9	Rh4O9.1	C3/V5	0.37	0.27	>50
		Rh4O9.2	C3/V5	0.16	0.03	>50
		Rh4O9.3	C3/V5	1.23	1.25	>50
		Rh4O9.4	C3/V5	0.39	0.03	>50
		Rh4O9.5	C3/V5	0.24	0.17	>50
	12-046	Rh046.1	GH	0.61	0.23	>50
		Rh046.2	GH	1.61	0.42	>50
		Rh046.3	GH	0.021	0.80	>50
	12-137	Rh137.1	C3/V5	0.60	0.20	>50
		Rh137.2	Interface	0.26	>50	>50
		Rh137.3	FP	0.08	>50	0.02
		Rh137.4	C3/V5	0.06	0.08	>50
		Rh137.5	C3/V5	0.02	0.03	>50
		Rh137.6	V3	5.32	0.46	0.22
	12-143	Rh143.1	V1/V3	54.64	24.30	>100
	12M169	Rh169.1	C3/V5	1.05	2.46	>50
	11M088	Rh088.1	GH	0.27	0.11	>50
NON-INF	4O9	Rh4O9.6	C3/V5	0.21	0.13	>50
		Rh4O9.7	V1/V3	0.41	0.76	>50
		Rh4O9.8	V1/V3	0.12	0.35	>50
	12-046	Rh046.4	GH	0.61	0.04	>50
		Rh046.5	GH	0.78	0.35	>50
		Rh046.6	V1/V3	0.24	0.11	>50
		Rh046.7	V1/V3	0.21	1.44	>50
INF	11M088	Rh088.2	GH	0.18	0.01	>50
		Rh088.3	GH	0.16	0.004	>50
		Rh088.4	GH	0.43	0.05	>50
		Rh088.5	V1/V3	0.23	0.33	>50
		Rh088.6	GH	0.36	0.02	>50
		Rh088.7	C3/V5	0.25	0.04	>50
		Rh088.8	C3/V5	0.35	0.08	>50
		Rh088.9	GH	0.51	0.09	>50
		Rh088.10	C3/V5	0.33	0.25	>50
		Rh088.11	GH	0.12	0.99	>50
	12M169	Rh129.2	C3/V5	0.74	0.11	>50
		Rh129.3	C3/V5	0.83	0.56	>50
		Rh129.4	C3/V5	0.08	0.05	>50
NI	BZ31	RhBZ31.1	C3/V5	0.20	0.60	>50
		RhBZ31.2	C3/V5	0.31	0.10	>50
		RhBZ31.3	C3/V5	0.95	0.45	>50
	BZ05	RhBZ05.1	GH	0.41	0.06	>50
		RhBZ05.2	GH	0.26	0.13	>50
		RhBZ05.3	GH	0.63	2.18	>50
	BZ11	RhBZ11.1	GH	0.08	0.60	>50

Table S2. Immunogenetic table of all rhesus mAbs. Related to Figure 1.

mAb ID	Epitope	Animal	Timepoint	VH gene	VD gene	VJ gene	CDRH3 aa	CDRH3 length	VH SHM % (nt)	VL/VK gene	VJ gene	CDRL3 aa	CDRL3 Length	VL/VK SHM %(nt)
Rh084.1	C3/V5	12-084	week 25	IGHV3-128*01	IGHD2-10*01	IGHJ5*01	TRDCSSSSCSWTYSRFDV	18	5	IGKV3-7*01	IGKJ3*01	YQYYSGYT	8	4
Rh084.2	C3/V5	12-084	week 25	IGHV3-128*01	IGHD2-10*01	IGHJ5*01	TRDCSSSACSSRYSRFDV	18	6	IGKV3-7*01	IGKJ3*01	YQYFSGYT	8	5
Rh084.3	C3/V5	12-084	week 25	IGHV3-128*01	IGHD2-10*01	IGHJ5*01	TRDCSSSSCSSRYSRFDV	18	6	IGKV3-7*01	IGKJ2*01	YQYFSGYT	8	4
Rh4O9.1	C3/V5	4O9	week 68	IGHV4-79*01	IGHD6-38*01	IGHJ4*01	ARAPTGS	7	6	IGKV1-25*01	IGKJ3*01	LQYFSLFT	8	7
Rh4O9.2	C3/V5	4O9	week 68	IGHV4-79*01	IGHD6-36*01	IGHJ4*01	ARAPTGN	7	10	IGKV1-25*01	IGKJ3*01	LQYFDLFT	8	7
Rh4O9.3	C3/V5	4O9	week 68	IGHV4-79*01	IGHD5-27*01	IGHJ4*01	ARGPTGN	7	9	IGKV1-25*01	IGKJ3*01	QQYFNFFS	8	7
Rh4O9.4	C3/V5	4O9	week 68	IGHV4-79*01	IGHD5-27*01	IGHJ4*01	VRGPTGN	7	9	IGKV1-25*01	IGKJ3*01	QQYFDLVT	8	7
Rh4O9.5	C3/V5	4O9	week 68	IGHV4-79*01	IGHD1-5*01	IGHJ4*01	ARSPTGN	7	9	IGKV1-25*01	IGKJ3*01	LQYFDLFT	8	5
Rh4O9.6	C3/V5	4O9	week 76	IGHV4-79*01	IGHD2-11*01	IGHJ4*01	ARTPTGD	7	9	IGKV1-25*01	IGKJ3*01	LQYFSLFT	8	6
Rh4O9.7	V1/V3	4O9	week 76	IGHV3-103*01	IGHD3-17*01	IGHJ4*01	ATTLGDYYVD	10	7	IGLV1-75*01	IGLJ3*01	AAWDDNLNGRV	11	4
Rh4O9.8	V1/V3	4O9	week 76	IGHV3-103*01	IGHD3-15*01	IGHJ4*01	TRTLGDYYLD	10	8	IGLV1-75*01	IGLJ3*01	AAWDDNLSGRI	11	4
Rh046.1	GH	12-046	week 26	IGHV4-149*01	IGHD4-24*01	IGHJ4*01	ARGGSSSLDF	10	10	IGKV1-50*01	IGKJ3*01	QQYNSDPFT	9	5
Rh046.2	GH	12-046	week 26	IGHV4-79*01	IGHD3-17*01	IGHJ4*01	AKGGEYAEY	9	11	IGKV1-50*01	IGKJ1*01	QQHKTYPWT	9	8
Rh046.3	GH	12-046	week 26	IGHV4-165*01	IGHD5-27*01	IGHJ4*01	ARGGQDFDY	9	13	IGKV1-50*01	IGKJ2*01	QQYNTYPYS	9	8
Rh046.4	GH	12-046	week 76	IGHV4-117*01	IGHD1-5*01	IGHJ4*01	ARGGSSSLDS	10	16	IGKV1-50*01	IGKJ3*01	HQYYRVPFT	9	7
Rh046.5	GH	12-046	week 76	IGHV4-149*01	IGHD1-8*01	IGHJ4*01	ARGGSEDFDS	10	12	IGLV1-55*01	IGLJ3*01	ASWDSRLNSVF	11	8
Rh046.6	V1/V3	12-046	week 76	IGHV3-127*01	IGHD2-11*01	IGHJ5*01	NTLPYCTGSRCYRGNRFDV	19	13	IGKV2-57*01	IGKJ1*01	MQGREFRT	8	9
Rh046.7	V1/V3	12-046	week 76	IGHV3-103*01	IGHD2-11*01	IGHJ5*01	NTLPYCTGGGCYRGNRFDV	19	11	IGKV2-57*01	IGKJ1*01	MNGLEFRT	8	9
Rh137.1	C3/V5	12-137	week 26	IGHV3-128*01	IGHD2-10*01	IGHJ5*01	TRDCSSSDCSSLFRRFDV	18	7	IGKV3-7*01	IGKJ2*01	YQYFSGYS	8	4
Rh137.2	interface	12-137	week 26	IGHV3-76*01	IGHD2-12*01	IGHJ4*01	AKDVPEYYSGGYPTFYFDF	19	11	IGKV1-49*01	IGKJ1*01	QQRNSHPPT	9	6
Rh137.3	FP	12-137	week 26	IGHV4-165*01	IGHD2-14*01	IGHJ4*01	ASGGYCSRGVCYAYFDS	17	8	IGKV1-50*01	IGKJ4*01	QQYNSAPLT	9	6
Rh137.4	C3/V5	12-137	week 76	IGHV3-128*01	IGHD2-10*01	IGHJ5*01	TRDCSSSDCSSSFRRFDV	18	7	IGKV3-7*01	IGKJ2*01	YQYFSGYT	8	7
Rh137.5	C3/V5	12-137	week 76	IGHV3-128*01	IGHD2-10*01	IGHJ5*01	TRDCSSSDCSSSFRRFDV	18	7	IGKV3-7*01	IGKJ2*01	YQYFSGYT	8	6
Rh137.6	V3	12-137	week 26	IGHV3-103*01	IGHD6-33*01	IGHJ5*02	SRAGRWYSSAWSSLDV	16	9	IGKV1-44*01	IGKJ3*01	LQGYSTPFT	9	7
Rh143.1	V1/V3	12-143	week 26	IGHV4-174*01	IGHD3-15*01	IGHJ4*01	TRDQDNYAYYYYFDC	15	2	IGKV1-25*01	IGKJ4*01	QQYNSLPLT	9	4
Rh169.1	C3/V5	12M169	week 28	IGHV3-28*01	IGHD6-39*01	IGHJ4*01	AVIAAATFVDY	11	5	IGKV1-51*01	IGKJ1*01	LQGLFTPWT	9	3
Rh169.2	C3/V5	12M169	week 86	IGHV1-58*01	IGHD3-15*01	IGHJ4*01	TRGVRHDYAYYYTNVDF	17	11	IGLV2-29*01	IGLJ1*01	SSFAGSHTFI	10	9
Rh169.3	C3/V5	12M169	week 86	IGHV3-28*01	IGHD6-39*01	IGHJ4*01	AAIAAATFIDY	11	11	IGKV1-44*01	IGKJ1*01	LQGFFTPWT	9	5
Rh169.4	C3/V5	12M169	week 86	IGHV1-58*01	IGHD3-15*01	IGHJ4*01	ARGVRHDYAEYYTTFDS	17	14	IGLV2-29*01	IGLJ1*01	SSYAGSNTFI	10	9
Rh088.1	GH	11M088	week 28	IGHV4-79*01	IGHD3-15*01	IGHJ4*01	STRMGET	7	11	IGKV3-11*01	IGKJ2*01	QQGSNWPYS	9	7
Rh088.2	GH	11M088	week 86	IGHV4-149*01	IGHD6-33*01	IGHJ3*01	ARKQDNFDF	9	11	IGKV1-50*01	IGKJ1*01	QQYYNEPWT	9	5
Rh088.3	GH	11M088	week 86	IGHV4-149*01	IGHD1-2*01	IGHJ3*01	ARKEDDFDF	9	12	IGKV1-50*01	IGKJ1*01	QQYYNDPWT	9	6
Rh088.4	GH	11M088	week 86	IGHV4-149*01	IGHD1-2*01	IGHJ3*01	ARKEDDFDF	9	11	IGKV1-50*01	IGKJ1*01	QQYNNDPWT	9	5
Rh088.5	V1/V3	11M088	week 86	IGHV3-172*01	IGHD2-10*01	IGHJ4*01	TTGEYFLHLDY	11	16	IGKV1-21*01	IGKJ3*01	LQHHTYPFT	9	12
Rh088.6	GH	11M088	week 86	IGHV4-149*01	IGHD1-2*01	IGHJ3*01	ARKEDDFDF	9	10	IGKV1-50*01	IGKJ1*01	QQYNNDPWT	9	5
Rh088.7	C3/V5	11M088	week 86	IGHV1-58*01	IGHD3-17*01	IGHJ3*01	ARGAVRGAYYYVTFDL	16	15	IGLV2-18*01	IGLJ1*01	SSYAGSGTFI	10	7
Rh088.8	C3/V5	11M088	week 86	IGHV1-58*01	IGHD1-4*01	IGHJ6*01	ARVVRTGLEQLLAGALDS	18	11	IGKV3-52*01	IGKJ2*01	QKYNDWPYS	9	5
Rh088.9	GH	11M088	week 86	IGHV4-149*01	IGHD6-33*01	IGHJ3*01	ARKQEDFDF	9	9	IGKV1-50*01	IGKJ1*01	QQYDNDPWT	9	8
Rh088.10	C3/V5	11M088	week 86	IGHV1-58*01	IGHD3-15*01	IGHJ4*01	ARATRDDNTYYYTHFDT	17	11	IGLV2-10*01	IGLJ1*01	CSYEASDTFI	10	6
Rh088.11	GH	11M088	week 86	IGHV3-50*01	IGHD2-11*01	IGHJ4*01	VRDQMEVVVRNSEIDY	16	7	IGKV3-9*01	IGKJ3*01	HQGNITPFT	9	7
RhBZ31.1	C3/V5	BZ31	week 86	IGHV3-128*01	IGHD2-10*01	IGHJ5*01	SRDCSSTDCSSSYSRFDV	18	3	IGKV3-7*01	IGKJ1*01	FQYFSGWT	8	3
RhBZ31.2	C3/V5	BZ31	week 86	IGHV3-128*01	IGHD2-10*01	IGHJ5*01	TRDCSSDFCSSSYNRFNV	18	4	IGKV3-7*01	IGKJ1*01	FQYYSGWT	8	3
RhBZ31.3	C3/V5	BZ31	week 86	IGHV3-128*01	IGHD2-10*01	IGHJ5*01	TRDCSSTSCSSSYSRFDV	18	4	IGKV3-7*01	IGKJ1*01	FQYFSGWT	8	4
RhBZ05.1	GH	BZ05	week 86	IGHV4-79*01	IGHD6-38*01	IGHJ4*01	VRDKGWD	7	12	IGKV3-11*01	IGKJ1*01	QQESNWPWT	9	6
RhBZ05.2	GH	BZ05	week 86	IGHV4-79*01	IGHD6-38*01	IGHJ4*01	ARDKGWD	7	9	IGKV3-11*01	IGKJ1*01	QQESNWPWT	9	8
RhBZ05.3	GH	BZ05	week 86	IGHV4-61*01	IGHD3-41*01	IGHJ1*01	ARDLDGGYNFWSGPPGYFEF	20	6	IGKV1-21*01	IGKJ2*01	LQHNTYPYS	9	6
RhBZ11.1	GH	BZ11	week 86	IGHV4-150*01	IGHD5-28*01	IGHJ4*01	AKERTTVQFDY	11	9	IGLV2-12*01	IGLJ1*01	SSYSSDDIYI	10	6

Table S3. Neutralization IC_{50} table of all rhesus mAbs against WT BG505 and MG505.A2 viruses, and variants with substitutions at the 241/289 GH, the C3/V5 epitope, the V1/V3 epitope, and the fusion peptide epitope, respectively. Related to Figure 2.

Epitope	Group	mAb ID	Maximum Neut (%)	WT IC50 (ug/mL)		241/289 GH		C3/V5			V1/V3		FP
				BG505	MG505.A2	BG505 N289	BG505 N241N289	BG505 TI357KT	MG505 KTII357TIIR	BG505 T465N	BG505 CTNN	BG505 Loop H3	BG505 N611A
C3/V5	IMM	Rh084.1	100	0.03	>50	0.25	0.56	>50	>50	0.03	0.22	0.35	0.09
	IMM	Rh084.2	100	0.09	>50	0.20	0.15	>50	7.88	0.30	0.07	0.11	0.02
	IMM	Rh084.3	100	0.10	>50	0.27	0.23	>50	1.94	0.32	0.40	0.10	0.02
	IMM	Rh4O9.1	100	0.07	>50	0.11	0.12	>50	1.57	0.27	0.10	0.04	0.09
	IMM	Rh4O9.2	100	0.05	>50	0.04	0.10	>50	1.11	0.03	0.04	0.04	0.07
	IMM	Rh4O9.3	100	0.15	>50	0.14	0.27	>50	>50	0.15	0.16	0.20	0.07
	IMM	Rh4O9.4	100	0.04	>50	0.05	0.03	>50	1.53	0.23	0.02	0.02	0.02
	IMM	Rh4O9.5	100	0.03	>50	0.08	0.26	>50	2.28	0.08	0.22	0.22	0.06
	NON-INF	Rh4O9.6	100	0.07	>50	0.12	0.05	3.97	1.80	0.35	0.04	0.06	0.09
	IMM	Rh137.1	100	0.08	>50	0.10	0.30	>50	14.21	5.10	0.18	0.10	0.13
	IMM	Rh137.4	100	0.02	>50	0.12	0.05	>50	1.69	0.17	0.03	0.04	0.08
	IMM	Rh137.5	100	0.91	>50	0.64	0.25	>50	6.54	3.39	0.19	0.25	0.38
	IMM	Rh169.1	100	0.28	>50	0.46	0.97	13.57	27.46	>50	1.43	0.55	0.44
	INF	Rh088.7	95	0.04	>50	0.02	0.08	>50	2.40	>50	0.05	0.11	0.04
	INF	Rh088.8	98	0.09	>50	0.07	0.13	>50	10.11	14.30	0.11	0.36	0.13
	INF	Rh088.10	95	0.08	>50	0.20	0.33	>50	15.51	>50	0.14	0.26	0.13
	INF	Rh129.2	98	0.06	>50	0.12	0.04	2.19	0.95	28.89	0.05	0.08	0.12
	INF	Rh129.3	100	0.10	>50	0.16	0.12	0.70	1.40	>50	0.06	0.10	0.16
	INF	Rh129.4	99	0.06	>50	0.64	0.11	>50	1.59	>50	0.04	0.06	0.10
	NI	RhBZ31.1	100	0.04	>50	0.05	0.27	>50	1.26	1.47	0.12	0.07	0.26
	NI	RhBZ31.2	100	0.02	>50	0.02	0.16	>50	0.79	0.42	0.09	0.14	0.04
	NI	RhBZ31.3	100	0.06	>50	0.32	3.91	>50	2.81	0.69	3.21	4.78	0.08
GH	IMM	Rh046.1	60	5.44	>50	>50	>50	>50	>50	12.97	37.22	>50	19.26
	IMM	Rh046.2	53	9.89	>50	>50	>50	>50	>50	29.38	>50	>50	42.05
	IMM	Rh046.3	50	14.36	>50	>50	>50	>50	>50	39.21	31.19	32.15	>50
	NON-INF	Rh046.4	51	19.96	>50	>50	>50	>50	>50	3.36	22.87	>50	32.18
	NON-INF	Rh046.5	50	17.54	>50	>50	>50	9.82	>50	31.13	28.75	35.45	19.51
	IMM	Rh088.1	54	11.03	>50	>50	>50	>50	>50	20.69	45.41	39.32	>50
	INF	Rh088.2	62	5.36	>50	>50	>50	24.45	>50	41.66	>50	27.76	1.64
	INF	Rh088.3	50	12.56	>50	>50	>50	>50	>50	>50	23.35	8.94	10.55
	INF	Rh088.4	53	8.69	>50	>50	>50	>50	>50	>50	25.13	2.52	24.09
	INF	Rh088.6	50	11.24	>50	>50	>50	>50	>50	7.46	5.25	4.28	14.73
	INF	Rh088.9	51	3.24	>50	>50	>50	10.47	>50	>50	44.36	45.51	2.07
	INF	Rh088.11	85	0.46	>50	18.42	>50	0.43	>50	4.29	1.50	0.97	1.09
	NI	RhBZ05.1	63	2.32	>50	>50	>50	32.30	>50	47.71	15.73	>50	33.41
	NI	RhBZ05.2	57	9.36	>50	>50	>50	42.68	>50	36.94	>50	>50	>50
	NI	RhBZ05.3	53	12.21	>50	16.01	>50	>50	>50	>50	24.81	>50	>50
	NI	RhBZ11.1	75	0.74	>50	0.24	>50	0.27	>50	15.07	27.75	20.05	1.33
	IMM	Rh143.1	70	8.42	36.78	46.35	>100	44.54	>100	36.53	>100	>100	38.22
V1/V3	NON-INF	Rh4O9.7	100	0.08	0.24	0.13	0.11	0.28	0.49	0.05	>50	>50	0.38
	NON-INF	Rh4O9.8	100	0.12	0.19	0.17	0.10	0.29	0.17	0.12	>50	>50	0.15
	NON-INF	Rh046.6	100	0.02	0.06	0.08	0.01	0.06	0.10	0.07	>50	>50	0.04
	NON-INF	Rh046.7	100	0.09	0.40	0.50	0.06	0.29	0.64	0.45	>50	>50	0.10
	INF	Rh088.5	100	0.04	0.10	0.03	0.03	0.09	0.14	0.12	>50	>50	0.06
Interface/FP	IMM	Rh137.2	95	3.04	2.98	5.76	23.95	2.10	2.33	4.37	38.51	32.79	0.17
	IMM	Rh137.3	34	>50	>50	>50	>50	>50	>50	>50	>50	>50	1.27
V3	IMM	Rh137.6	0	>50	>50	N/A	N/A	N/A	N/A	N/A	N/A	N/A	>50

N/A: not applicable

Table S4. Neutralization IC$_{50}$ (µg/mL) breadth of all rhesus mAbs including PGT121 as a control against a global panel of HIV isolates from multiple clades. Related to Figure 2.

	398F1	246F3	CNE55	CNE8	X2278	TRO11	BJOX2000	CH119	CE0217	CE1176	25710	X1632
Clade	A	AC	AE	AE	B	B	BC	BC	C	C	C	G
Rh084.1	>50	>50	>50	>50	>50	>50	>50	>50	>50	>50	>50	>50
Rh084.2	>50	>50	>50	>50	>50	>50	>50	>50	>50	>50	>50	>50
Rh084.3	>50	>50	>50	>50	>50	>50	>50	>50	>50	>50	>50	>50
Rh4O9.1	>50	>50	>50	>50	>50	>50	>50	>50	>50	>50	>50	>50
Rh4O9.2	>50	>50	>50	>50	>50	>50	>50	>50	>50	>50	>50	>50
Rh4O9.3	>50	>50	>50	>50	>50	>50	>50	>50	>50	>50	>50	>50
Rh4O9.4	>50	>50	>50	>50	>50	>50	>50	>50	>50	>50	>50	>50
Rh4O9.5	>50	>50	>50	>50	>50	>50	>50	>50	>50	>50	>50	>50
Rh046.1	>50	>50	>50	>50	>50	>50	>50	>50	>50	>50	>50	>50
Rh046.2	>50	>50	>50	>50	>50	>50	>50	>50	>50	>50	>50	>50
Rh046.3	>50	>50	>50	>50	>50	>50	>50	>50	>50	>50	>50	>50
Rh137.1	>50	>50	>50	>50	>50	>50	>50	>50	>50	>50	>50	>50
Rh137.2	>50	>50	>50	>50	>50	>50	>50	>50	>50	>50	>50	>50
Rh137.3	>50	>50	>50	>50	>50	>50	>50	>50	>50	>50	>50	>50
Rh137.4	>50	>50	>50	>50	>50	>50	>50	>50	>50	>50	>50	>50
Rh137.5	>50	>50	>50	>50	>50	>50	>50	>50	>50	>50	>50	>50
Rh137.6	18.00	>50	>50	>50	>50	>50	>50	>50	>50	>50	>50	>50
Rh143.1	>50	>50	>50	>50	>50	>50	>50	>50	>50	>50	>50	>50
Rh169.1	>50	>50	>50	>50	>50	>50	>50	>50	>50	>50	>50	>50
Rh088.1	>50	>50	>50	>50	>50	>50	>50	>50	>50	>50	>50	>50
Rh4O9.6	>50	>50	>50	>50	>50	>50	>50	>50	>50	>50	>50	>50
Rh4O9.7	>50	>50	>50	>50	>50	>50	>50	>50	>50	>50	>50	>50
Rh4O9.8	>50	>50	>50	>50	>50	>50	>50	>50	>50	>50	>50	>50
Rh046.4	>50	>50	>50	>50	>50	>50	>50	>50	>50	>50	>50	>50
Rh046.5	>50	>50	>50	>50	>50	>50	>50	>50	>50	>50	>50	>50
Rh046.6	>50	>50	>50	>50	>50	>50	>50	>50	>50	>50	>50	>50
Rh046.7	>50	>50	>50	>50	>50	>50	>50	>50	>50	>50	>50	>50
Rh088.2	>50	>50	>50	>50	>50	>50	>50	>50	>50	>50	>50	>50
Rh088.3	>50	>50	>50	>50	>50	>50	>50	>50	>50	>50	>50	>50
Rh088.4	>50	>50	>50	>50	>50	>50	>50	>50	>50	>50	>50	>50
Rh088.5	>50	>50	>50	>50	>50	>50	>50	>50	>50	>50	>50	>50
Rh088.6	>50	>50	>50	>50	>50	>50	>50	>50	>50	>50	>50	>50
Rh088.7	>50	>50	>50	>50	>50	>50	>50	>50	>50	>50	>50	>50
Rh088.8	>50	>50	>50	>50	>50	>50	>50	>50	>50	>50	>50	>50
Rh088.9	>50	>50	>50	>50	>50	>50	>50	>50	>50	>50	>50	>50
Rh088.10	>50	>50	>50	>50	>50	>50	>50	>50	>50	>50	>50	>50
Rh088.11	>50	>50	>50	>50	>50	>50	>50	>50	>50	>50	>50	>50
Rh169.2	>50	>50	>50	>50	>50	>50	>50	>50	>50	>50	>50	>50
Rh169.3	>50	>50	>50	>50	>50	>50	>50	>50	>50	>50	>50	>50
Rh169.4	>50	>50	>50	>50	>50	>50	>50	>50	>50	>50	>50	>50
RhBZ31.1	>50	>50	>50	>50	>50	>50	>50	>50	>50	>50	>50	>50
RhBZ31.2	>50	>50	>50	>50	>50	>50	>50	>50	>50	>50	>50	>50
RhBZ31.3	>50	>50	>50	>50	>50	>50	>50	>50	>50	>50	>50	>50
RhBZ05.1	>50	>50	>50	>50	>50	>50	>50	>50	>50	>50	>50	>50
RhBZ05.2	>50	>50	>50	>50	>50	>50	>50	>50	>50	>50	>50	>50
RhBZ05.3	>50	>50	>50	>50	>50	>50	>50	>50	>50	>50	>50	>50
RhBZ11.1	>50	>50	>50	>50	>50	>50	>50	>50	>50	>50	>50	>50
PGT121	0.003	>50	>50	>50	0.02	0.001	0.07	0.02	0.01	0.02	0.01	>50
