# Supplementary material for: Mapping Neutralizing Antibody Epitope Specificities to an HIV Env Trimer in Immunized and in Infected Rhesus Macaques
Source: Cell Rep. 2020 Sep 8;32(10):108122. doi: 10.1016/j.celrep.2020.108122 (PMC7487785; doi:10.1016/j.celrep.2020.108122)
Supplement: Document S2. Article plus Supplemental Information [file mmc2.pdf]

Cell Reports

Article

Mapping Neutralizing Antibody Epitope Specificities to an HIV Env Trimer in Immunized and in Infected Rhesus Macaques

Graphical Abstract

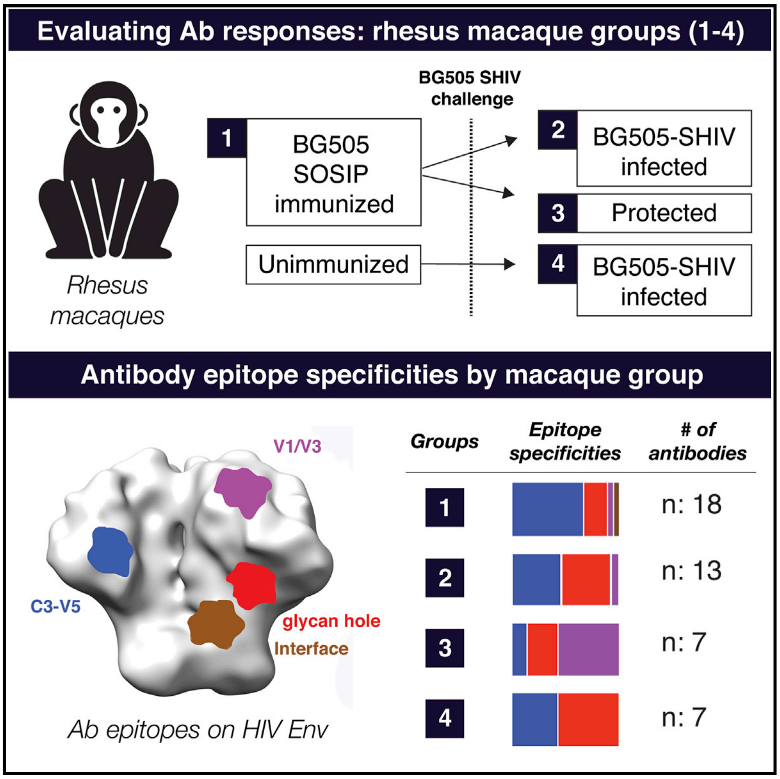

Authors

Fangzhu Zhao, Collin Joyce, Alison Burns, ..., Andrew B. Ward, Dennis R. Burton, Devin Sok

Correspondence

burton@scripps.edu (D.R.B.), dsok@iavi.org (D.S.)

In Brief

Zhao et al. report the isolation and characterization of neutralizing antibodies from BG505 SHIV-infected and BG505 SOSIP-immunized macaques. Antibody responses between immunization and natural infection are similar and protective epitope specificities mapped. A common neutralizing antibody lineage (public clonotype) is identified across several macaques targeting an immunodominant epitope.

Highlights

- SHIV-infected and SOSIP-immunized macaques elicit similar neutralizing antibodies
- Four neutralizing epitope specificities are found in BG505 SOSIP-immunized macaques
- Antibodies to V1/V3 and C3/V5 are potently neutralizing and contribute to protection
- Public clonotype is identified against immunodominant C3/V5 epitope

Zhao et al., 2020, Cell Reports *32*, 108122
September 8, 2020 © 2020 The Author(s).
https://doi.org/10.1016/j.celrep.2020.108122

Cell Reports

CellPress
OPEN ACCESS

Article

Mapping Neutralizing Antibody Epitope Specificities to an HIV Env Trimer in Immunized and in Infected Rhesus Macaques

Fangzhu Zhao,[1,2,3] Collin Joyce,[1,2,3] Alison Burns,[1,2,3] Bartek Nogal,[5] Christopher A. Cottrell,[5] Alejandra Ramos,[1,2,3,4] Trevor Biddle,[1,2,3] Matthias Pauthner,[1,2,3] Rebecca Nedellec,[1,2,3] Huma Qureshi,[1,2,3,4] Rosemarie Mason,[7] Elise Landais,[1,2,3,4] Bryan Briney,[1,2,6] Andrew B. Ward,[2,3,5] Dennis R. Burton,[1,2,3,8,*] and Devin Sok[1,2,3,4,9,*]
[1]Department of Immunology and Microbiology, The Scripps Research Institute, La Jolla, CA 92037, USA
[2]Center for HIV/AIDS Vaccine Development (CHAVD), The Scripps Research Institute, La Jolla, CA 92037, USA
[3]IAVI Neutralizing Antibody Center, The Scripps Research Institute, La Jolla, CA 92037, USA
[4]IAVI, New York, NY 10004, USA
[5]Department of Integrative Structural and Computational Biology, The Scripps Research Institute, La Jolla, CA 92037, USA
[6]Center for Viral Systems Biology, The Scripps Research Institute, La Jolla, CA 92037, USA
[7]Vaccine Research Center, National Institutes of Health, Bethesda, MD 20892, USA
[8]Ragon Institute of Massachusetts General Hospital, Massachusetts Institute of Technology, and Harvard, Cambridge, MA 02114, USA
[9]Lead Contact
*Correspondence: burton@scripps.edu (D.R.B.), dsok@iavi.org (D.S.)
https://doi.org/10.1016/j.celrep.2020.108122

SUMMARY

BG505 SOSIP is a well-characterized near-native recombinant HIV Envelope (Env) trimer that holds promise as part of a sequential HIV immunogen regimen to induce broadly neutralizing antibodies (bnAbs). Rhesus macaques are considered the most appropriate pre-clinical animal model for monitoring antibody (Ab) responses. Accordingly, we report here the isolation of 45 BG505 autologous neutralizing antibodies (nAbs) with multiple specificities from SOSIP-immunized and BG505 SHIV-infected rhesus macaques. We associate the most potent neutralization with two epitopes: the C3/V5 and V1/V3 regions. We show that all of the nAbs bind in close proximity to known bnAb epitopes and might therefore sterically hinder elicitation of bnAbs. We also identify a "public clonotype" that targets the immunodominant C3/V5 nAb epitope, which suggests that common antibody rearrangements might help determine humoral responses to Env immunogens. The results highlight important considerations for vaccine design in anticipation of results of the BG505 SOSIP trimer in clinical trials.

INTRODUCTION

The elicitation of broadly neutralizing antibodies (bnAbs) remains a key strategy in generating a protective HIV vaccine. These antibodies are capable of neutralizing a large diversity of HIV isolates by targeting relatively conserved epitopes on the HIV Env glycoprotein, which is the sole target of nAbs and comprises a trimer of heterodimers, gp120 and gp41. Many groups in the field are exploring different approaches to the elicitation of bnAbs, including lineage-based design and germline targeting, to direct and guide the immune system to affinity mature specific antibody responses (Andrabi et al., 2018; Briney et al., 2016; Jardine et al., 2015, 2016; Sok et al., 2016a; Xu et al., 2018). Notwithstanding, these sequential immunization approaches will likely conclude with a native-like trimer to elicit antibodies that recognize the native Env trimer present on HIV (Burton, 2019). To this end, the field has generated a number of native-like trimer designs that have been characterized and tested in different animal models (Bradley et al., 2016; Havenar-Daughton et al., 2016; Sanders and Moore, 2017; Sanders et al., 2015; de Taeye et al., 2016). The best described and characterized among these is BG505 SOSIP.664 gp140 (BG505 SOSIP) (Sanders et al., 2013), which was the first trimer reported to reliably elicit potent autologous nAb responses in animal models by vaccination (Sanders et al., 2015).

Since it was first reported, BG505 SOSIP has been used to immunize a number of different animals, including rabbits, guinea pigs, cows, mice, humanized mice, and rhesus macaques (Feng et al., 2016; Hu et al., 2015; Pauthner et al., 2017; Sanders et al., 2015; Sok et al., 2017). Interestingly, the humoral responses vary considerably between the different animals. In mice and humanized mice, for example, neutralization was not detected, although robust binding titers were reported with the base of the trimer identified as immunodominant (Hu et al., 2015). Potent autologous nAbs were reliably reported in rabbits and guinea pigs, with the "glycan hole" centered on positions N241 and N289 as immunodominant in rabbits (Bianchi et al., 2018; Klasse et al., 2018; McCoy et al., 2016) and the

This is an open access article under the CC BY license (http://creativecommons.org/licenses/by/4.0/).

C3/V4 epitope region as immunodominant in guinea pigs (Lei et al., 2019). In rhesus macaques, the nAb titers were lower than those observed in rabbits and guinea pigs and serum mapping studies identified the C3/V5 epitope region as immunodominant (Cirelli et al., 2019; Klasse et al., 2018; Sanders et al., 2015). Finally, in cows, immunization with the BG505 SOSIP alone resulted in rapid, broad, and potent nAb responses, which were directed to the CD4 binding site (Sok et al., 2017). These different immune responses highlight the important role of antibody repertoires in shaping immunodominant responses to the same trimer immunogen. Importantly, monoclonal antibodies (mAbs) have been isolated and characterized for all of the animals yielding nAb responses but only few for rhesus macaques (Cottrell et al., 2020). MAbs provide a more detailed view of the epitopes targeted on HIV Env, and comparison of their sequences might reveal common signatures that contribute to immunogenicity. Additionally, first-in-human phase I trials have initiated for BG505 SOSIP at three clinical trial sites, two in the United States, and one in Kenya. The availability of mAbs from immunized rhesus macaques might provide early insight to what responses are predicted in humans given the close similarities between the macaque and human immunoglobulin (Ig) loci (Sundling et al., 2012). Additionally, once data from the human trial are available, a comparison of serum immune responses and mAbs will determine which animal model is the most predictive of immune responses observed in humans and therefore the one most appropriate for further study.

In this study, we report the isolation and characterization of monoclonal nAbs from BG505 SOSIP-immunized Indian rhesus macaques. This work follows immunization of rhesus macaques with BG505 SOSIP that generated varying serum levels of autologous nAbs (Pauthner et al., 2017). Six animals with high serum nAb titers, six with low serum nAb titers, and six unimmunized animals were subjected to repeated low-dose virus challenge with chimeric simian-human immunodeficiency virus (SHIV) with the BG505 envelope virus sequence (Pauthner et al., 2019). Since nAb titers declined over the period of challenges, the approximate titers at which animals became infected could be determined. In the high nAb titer group, two animals with serum nAb titers >1:500 were uninfected throughout the course of the experiment, while four animals became infected as their nAb titers decayed below this threshold. All six of the low nAb titers became rapidly infected in one or two challenges as did the unimmunized control animals. Among the high nAb titer animals that were infected following challenge, increases in nAb titers were observed after infection suggesting either recall of previously primed memory B cell responses or elicitation of new B cell responses that augment previous responses. We were interested in determining the extent of differences in the epitope(s) recognized by protected animals compared to those that became infected. Additionally, we were interested in determining whether increases in nAb titers post-challenge of the high nAb-titer animals who became infected were due to recall of Env trimer immunogen-primed responses or whether *de novo* responses were elicited. Accordingly, mAbs were isolated from rhesus macaques: (1) post-immunization with BG505 SOSIP, the high titer nAb group, but pre-SHIV challenge (IMM), (2) post-SHIV infection, the high nAb titer group, in which animals became infected (INF), (3) post SHIV challenge, high nAb titer group, in which animals did not become infected (NON-INF), and (4) unimmunized and SHIV infected (NI, natural infection).

We report the isolation of 45 nAbs from 9 rhesus macaques that target three nAb specificities on BG505 Env as well as one non-neutralizing antibody to BG505 and one antibody that modestly neutralizes virus 398F1 but not BG505. Among these 45 nAbs, we observed a common VDJ rearrangement from three individual rhesus macaques that all target the immunodominant C3/V5 epitope and identified this rearrangement in other macaques not involved in this study. The results provide a comprehensive assessment of nAb responses to the BG505 Env in rhesus macaques under differing conditions and serve as a valuable resource for comparison to upcoming human clinical trial data.

RESULTS

High-Throughput Isolation of nAbs to BG505 through B Cell Activation and Functional Screens

To evaluate the immunogenicity of BG505 Env and the corresponding nAb specificities, we isolated mAbs from BG505 SOSIP-immunized and $SHIV_{BG505}$-infected macaques in the four groups listed above. In the high nAb titer group, vaccine-induced mAbs were isolated after the second (week 24) or third (week 64) immunization boost before SHIV challenge (IMM) (Figure 1A). All animals were then repeatedly challenged with $SHIV_{BG505}$ beginning at week 68. Of the IMM group, two animals, 4O9 and 12-046, remained protected after 12 repeat virus challenges. Their serum nAb titers slowly decreased and then plateaued, indicating no secondary Ab response and consistent with the finding of complete protection from infection (Pauthner et al., 2019). We isolated mAbs at week 76 to investigate the nAbs epitopes that might contribute to protection (NON-INF) (Figure 1A). Protection was reported for animals 12-137, 11M088, and 12M169 after 6 challenges. These animals, however, became infected at week 8, week 14, and week 16 after virus challenge, respectively. Accordingly, we isolated mAbs at week 86 from 11M088 and 12M169 at their peak nAb titers post-infection (INF). In the unimmunized group, we isolated mAbs from infected naive animals BZ31, BZ05, and BZ11 at week 86 after challenge, as they developed medium to high serum BG505 nAb titers (NI) (Figure 1A).

A hybrid of an antigen-selection and high-throughput functional screening strategy was used to isolate BG505 mAbs from peripheral blood mononuclear cells (PBMCs) (Huang et al., 2013). $CD20^{+}IgM^{-}IgG^{+}$ memory B cells were sorted for binding to a BG505 SOSIP.664 probe conjugated with two different fluorophores (Figure S1A). A BG505 Env dual-positive population was single-cell sorted and expanded *in vitro* using a cocktail of cytokines and a fibroblast feeder layer expressing CD40 ligand (Huang et al., 2013). Supernatants from cultured B cells were harvested and screened for neutralization of BG505 pseudovirus. Immunoglobulin heavy-chain (IgH) and light-chain (IgL) genes were rescued from positive hits by reverse transcriptase PCR (RT-PCR) and PCR and then cloned into antibody expression vectors and expressed using a mammalian production system (Huang et al., 2013; Tiller et al., 2008). As shown previously, the high nAb titer animals developed sporadically heterologous serum neutralization responses against a

CellPress
OPEN ACCESS

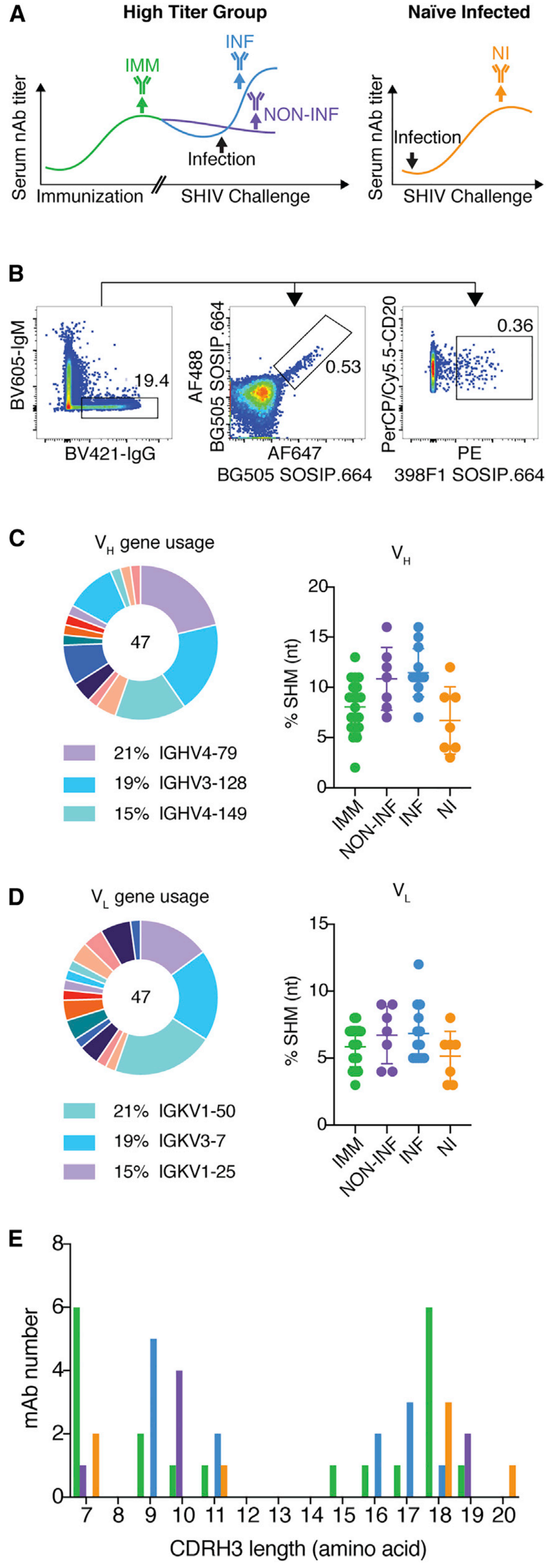

Figure 1. Isolation of BG505 Env mAbs from Immunized, Immunized/Infected, Immunized/Non-infected, and Infected NHPs

(A) Schema illustrating the 4 different groups of NHPs from which mAbs were isolated. In the high nAb titer group, mAbs were isolated post-immunization (IMM, green) and, after SHIV challenge, from two protected animals (NON-INF, purple) and from two non-protected animals (INF, blue). MAbs were also isolated from unimmunized SHIV- infected (natural infection) animals (NI, orange).

(B) FACS layout from rhesus PBMCs, in which population is gated on lymphocytes/singlets/$CD3^{-}CD4^{-}CD8^{-}CD14^{-}CD20^{+}$ B cells. $IgM^{-}IgG^{+}$BG505 SOSIP.664^{2+} or 398F1.664 $SOSIP^{+}$ memory B cells were single-cell sorted.

(C and D) 47 BG505 Env-specific paired heavy-chain sequences (C) and 47 light-chain sequences (D) were obtained. Heavy- and light-chain somatic hypermutation rates at the nucleotide level among mAbs isolated post-immunization (IMM), post-immunization/infection (INF), post-immunization without infection (NON-INF), and from natural infection (NI) are shown. Error bars for (C) and (D) represent standard deviation.

(E) The amino acid CDRH3 lengths are shown for isolated mAbs, color-coded based on group (IMM, green; NON-INF, purple; INF, blue; NI, orange).

global panel of viruses after three immunizations (deCamp et al., 2014; Pauthner et al., 2019). Since most vaccinated NHPs only displayed high cross-neutralization serum antibody titers to 398F1, we also used 398F1 SOSIP.664 as a second probe to screen and isolate heterologous nAbs (Figure 1B).

We sorted 10,889 Ag-specific memory B cells into 384-well plates, among which 3,195 wells (29% efficiency) expressed detectable IgG in cell-culture supernatants. In the micro-neutralization screening, 63 wells tested positive for neutralization against BG505 virus (2% of IgG positive wells) (Figure S1B). We subsequently cloned and expressed 20 mAbs from 6 high-titer animals in the IMM group, 13 mAbs from 2 animals in the INF group, 7 mAbs from 2 protected animals in the NON-INF group, and 7 mAbs from 3 unimmunized animals in the NI group. Each mAb was named by the animal identifier (Rh084, Rh4O9, etc.) and then followed by numerical order for each animal. Binding to BG505 SOSIP was confirmed for all 47 mAbs, of which 45 tested positive for binding to BG505 gp120 (Table S1). Two mAbs bound to 398F1 SOSIP, one targeting the fusion peptide and the other targeting the V3 loop, which also both bound to BG505 SOSIP. mAbs were characterized for Ig gene usage, somatic hypermutation (SHM) levels and distribution of complementarity determining region 3 (CDR3) length (Table S2). We annotated Ig sequences with an improved rhesus macaque germline database (Cirelli et al., 2019). V_H 4-79 and V_H 3–128 segments were frequently used (21% and 19%, respectively) while light-chain genes were predominantly V_k 1–50 and V_k 3–7 (21% and 19%, respectively) (Figures 1C and 1D). The average SHM rates at the nucleotide level among antibody heavy chains and light chains were 9.1% and 6.2%, respectively (Figures 1C and 1D). mAbs isolated from immunized animals that were subsequently infected with $SHIV_{BG505}$ challenge virus showed increased SHM compared to mAbs isolated after trimer immunization alone, suggesting additional selection and affinity maturation of pre-existing vaccine-induced mAbs in germinal centers following virus challenge (Havenar-Daughton et al., 2017). Interestingly, around 48% of the mAbs displayed CDRH3s less than 11 amino acids (aa) length (Figure 1E), while the average in macaques is approximately 14 aa (Link et al., 2005). The short CDRH3 length indicates that the autologous nAb epitopes on BG505 Env are likely more accessible than the epitopes of

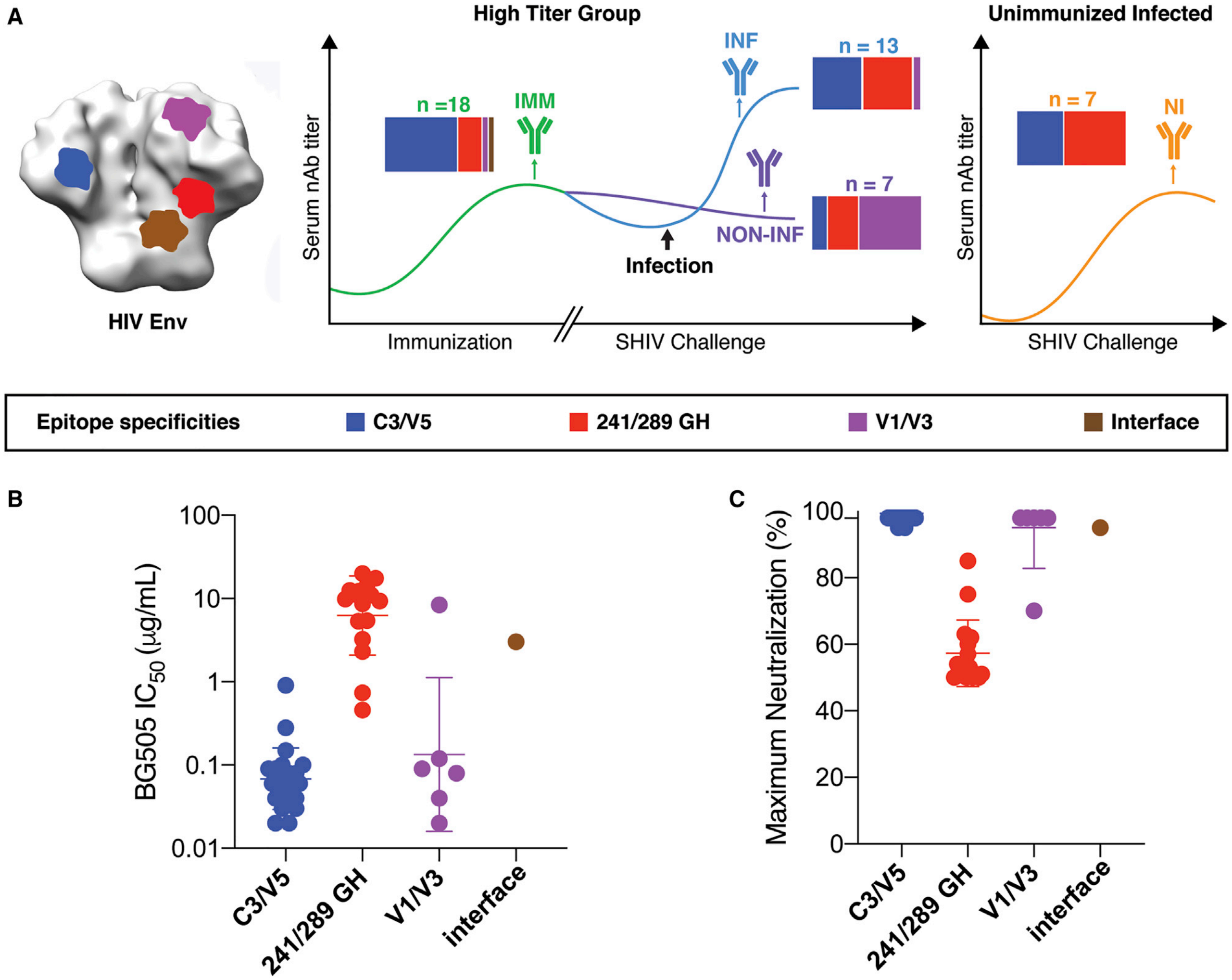

Figure 2. Neutralization Activities and Specificities of Monoclonal Antibodies Isolated following Env Vaccination and Natural Infection

(A) Left: NS-EM image of HIV-1 Env trimer with regions recognized by nAbs represented by colored patches. Right: horizontal bar chart of nAb specificities to BG505 Env trimer in the mAb isolation scheme. Blue: C3/V5; red: 241/289 glycan hole (GH); purple: V1/V3; brown: gp120-gp41 interface.

(B) Neutralization IC_{50} potency of nAbs to BG505 pseudovirus, listed by epitope specificity. Assays were run in duplicate. Error bars represent standard deviation.

(C) Maximum percent neutralization of nAbs to BG505 pseudovirus at the highest antibody concentrations (50 μg/mL), listed by epitope specificity. Error bars represent standard deviation.

bnAbs and do not require the long CDRH3s typically associated with penetration of the glycan shield (Doria-Rose et al., 2014; Walker et al., 2009, 2011).

Rhesus Macaques Develop Three Major Autologous nAb Specificities in Response to Recombinant BG505 SOSIP Trimer Immunization

We next tested the 47 mAbs for neutralization of autologous BG505 pseudovirus. Of these, 45 rhesus mAbs showed neutralization activity against BG505 pseudovirus with 50% inhibitory concentration values (IC_{50}) ranging from 0.02 to 20 μg/mL (Table S3), and one showed modest neutralizing activity against virus 398F1 (Table S4) but not to BG505 virus indicating serum neutralization breadth could be due to combinations of strain-specific neutralizing antibodies (Nogal et al., 2020; Pauthner et al., 2017). Subsequently, to assess the epitope specificities of isolated mAbs, we mapped their neutralization sensitivities to different BG505 virus mutants. The rhesus mAbs targeted diverse specificities on the BG505 Env trimer including the N241/N289 glycan hole, the C3/V5 region, the V1/V3 region, and the gp120-gp41 interface, among which the C3/V5 and N241/N289 glycan hole epitopes were immunodominant among vaccinated and naive infected rhesus macaques (Figure 2A).

In general, nAbs to C3/V5 and V1/V3 neutralized BG505 potently and completely with maximum neutralization plateaus reaching 100%. C3/V5-targeting mAbs neutralized BG505 at a median IC_{50} of 0.06 μg/mL and completely lost neutralization activities against a BG505 mutant with the ^{357}TIIR^{360} residues on the C3 loop mutated to TI357KT (McCoy et al., 2016; Figure 2B; Table S3). Most rhesus mAbs to the V1/V3 loop neutralized

CellPress
OPEN ACCESS

BG505 potently (median IC_{50} of 0.08 μg/mL), whereas one isolated mAb displayed poor neutralization potency. BG505 pseudovirus with glycan sequons (N-X-T/S, where X can be any amino acid but proline) shifted from N133 and N137 to N134 and N139 disrupted neutralization of V1/V3 epitope targeting nAbs (Klasse et al., 2018; Table S3). Rh137.2, the only nAb that targeted the gp120-gp41 interface, neutralized BG505 completely but moderately with an IC_{50} of 3.04 μg/mL. This nAb displayed a decrease in neutralization potency against BG505 with the N88 glycan sequon deleted and increased potency against BG505 N611A (Figure S2A). In contrast, most mAbs against the N241/N289 glycan hole displayed incomplete neutralization of BG505, with 50% to 70% of maximum neutralization activity at the highest concentration (50 μg/mL) (Figure 2C). Interestingly, such incomplete neutralization was not observed for rabbit glycan hole-targeting mAbs, which fully neutralized BG505 (McCoy et al., 2016). These differences might be due to differences in CDR loop lengths, where the average CDRH3 length for rabbit nAbs is 13–15 aa compared to 10 aa for the rhesus nAbs. Additionally, rhesus nAbs to the glycan hole had weak neutralization potency (median IC_{50} of 9.63 μg/mL) relative to C3/V5 and V1/V3 nAbs (Figure 2B).

To determine whether the mAbs had any neutralization breadth, we next tested all isolated mAbs on a global panel of viruses from multiple clades and all were found to be largely strain-specific for BG505 virus (Table S4; Seaman et al., 2010). With the partial exception of the interface-targeting mAb Rh137.02, which neutralized MG505.A2 moderately, only the V1/V3-targeting antibodies potently neutralized MG505.A2, the maternal virus closely associated with BG505 and having an identical V1 sequence (Table S3). The V1/V3-targeting nAbs completely lost neutralization ability when the V1 loop of MG505.A2 was replaced with the V1 loop of MG505.H3 (Figure S2B; Klasse et al., 2018).

None of the nAbs bound heterologous Env trimers on the global indicator panel except Rh137.3, which showed binding to a few Env trimers (Figure S2C). Rh137.3 targets the fusion peptide (FP) on BG505 SOSIP but does not neutralize BG505 virus. Interestingly, Rh137.3 neutralizes BG505 virus with the glycan sequon at N611 removed (N611A) but not 398F1 N611A despite 398F1 sharing eight identical FP residues with BG505 (Figure S2A). To understand this discrepancy, we performed negative stain electron microscopy (EMPEM), which shows that Rh137.3 binds the FP epitope region of BG505 SOSIP at a different angle of approach from VRC34.01, the human FP-targeting HIV bnAb (Figure S2D; Kong et al., 2016). Such different orientation of binding may explain the limited breadth of neutralization of this mAb. Serum neutralization breadth and more recent electron microscopy polyclonal epitope mapping studies do, however, indicate the presence of FP-targeting antibodies capable of binding and neutralizing 398F1 (Nogal et al., 2020; Pauthner et al., 2017).

Multiple Specificities against Potent Epitopes Observed in Fully Protected Animals

After determining mAb specificities, we next evaluated the corresponding rhesus macaque sera at the time of SHIV challenge. We elected to focus on the C3/V5 and the V1/V3 specificities as the isolated nAbs to these sites potently neutralized BG505 with neutralization plateaus reaching 100%. Serum neutralization specificities were mapped for all 6 rhesus macaques that comprise the high nAb-titer group, which had received 12 (two sets of 6) repeated low-dose challenges with $SHIV_{BG505}$ (Nogal et al., 2020; Pauthner et al., 2017).

We first mapped neutralizing epitope specificities for the two protected animals. To map the C3/V5 serum epitope specificity, we compared serum neutralization activity for BG505 and its corresponding TI357KT mutant (Figure 3A). To map the V1/V3 region specificity, we compared serum neutralization against MG505.A2, which has a conserved BG505 V1 epitope with MG505.A2 loop H3 (mutations in the V1 loop that abrogate neutralization activity) (Figure 3B). For the first 6 challenges, the data indicate that the main nAb specificity for both protected macaques is to the V1/V3 epitope. These V1/V3 titers decreased over time for both animals with a total loss of this titer for animal 4O9 by week 14 and sustained titers for animal 12-046. Interestingly, for animal 4O9, as nAb titers to the V1/V3 epitope decreased, nAb titers to the C3/V5 epitope increased, particularly for the second set of challenges (Figure 3A). We also confirmed that protection in these animals was likely antibody mediated by delivering an anti-CD8a mAb to deplete CD8 T and NK cells, which did not result in outgrowth of virus, indicating the animals were indeed fully protected throughout and following virus challenge (Figures S3A–S3C).

We next evaluated the serum neutralizing specificities for the non-protected animals. Determination of neutralization against BG505 and the corresponding TI357KT mutant (Figure 3C) revealed that the dominant nAb epitope is C3/V5. Non-protected animals barely developed any V1/V3 response following boost immunization or following challenge (Figure 3D). Only animal 11M088 developed modest neutralizing responses to MG505.A2 prior to challenge, indicating a V1/V3-directed response, but these titers declined quickly after challenge. Animals 11M088 and 12M169 began to develop high V1/V3 nAb titers after they became infected, suggesting redirected nAb specificities after exposure to SHIV infection (Nogal et al., 2020).

BG505 serum nAb titers have been shown to strongly correlate with protection from SHIV challenge, but we were also interested in determining whether elicitation of nAbs to multiple specificities might have an additive or synergistic protective effect. First, we evaluated mAbs for neutralization against the putative escape viruses reported from this study. Viral *env* sequencing from animal 12-137 and 12-143 after infection revealed putative escape mutations at K168E and R192K residues on the V2 loop as well as G354R and N356K residues on the C3 loop, which flanks the N355 glycan (Pauthner et al., 2019). As expected, nAbs targeting the C3/V5 loop failed to neutralize BG505 with G354R or N356K mutations, while nAbs to V1/V3, the N241/N289 glycan hole, and the gp120-gp41 interface retained moderate to potent neutralization activities for both viruses (Figure 3E). The results suggest that the immunodominant C3/V5 autologous nAb responses initially selected for virus escape against this epitope, but that the availability of a second nAb specificity was able to protect against this escape virus.

We next evaluated whether a combination of V1/V3 and C3/V5 nAbs might result in enhanced neutralization or enhanced

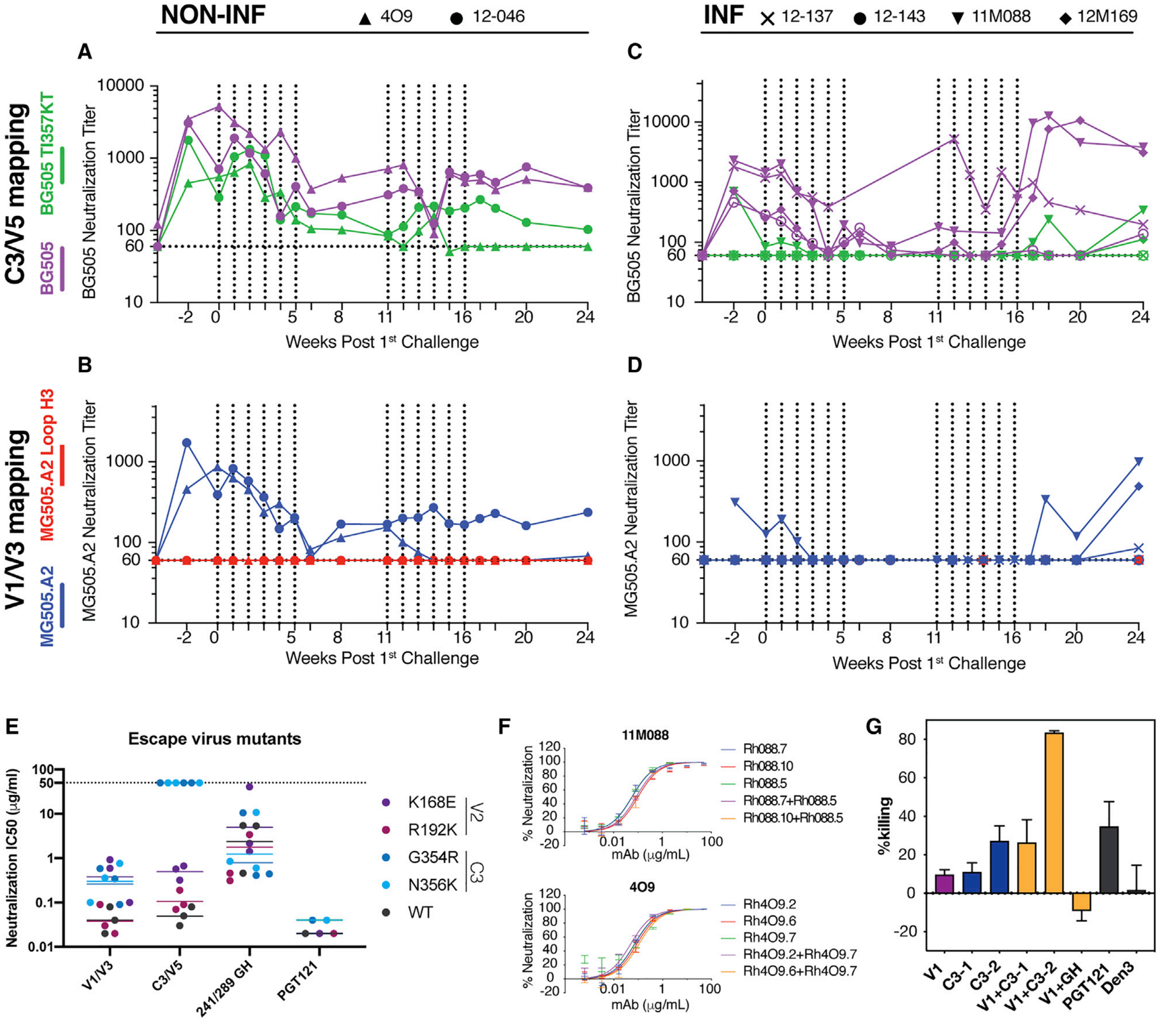

Figure 3. Mapping the nAb Specificities Associated with Protection in BG505 SOSIP-Immunized NHPs

(A) Serum nAb titers against BG505 wild-type pseudovirus (purple) and BG505 TI357KT (green) pseudovirus, which is a C3/V5 epitope knockout mutant. Serum nAb ID_{50} were measured after each $SHIV_{BG505}$ challenge and as shown for protected animals 4O9 and 12-046. Vertical dashed lines represent for each challenge. The TI357KT substitution is expected to eliminate C3/V5-directed neutralization and therefore the difference between ID_{50}s shown in the figure reflects the likely contribution of that specificity to neutralization of wild-type $SHIV_{BG505}$. Assays were run in duplicate.

(B) Serum nAb ID_{50} titers were measured against MG505.A2 pseudovirus (blue) and V1-loop mutant virus MG505.A2 Loop H3 (red) for protected animals. Vertical dashed lines represent for each challenge.

(C) As for (A) but for the non-protected animals.

(D) As for (B) but for non-protected animals.

(E) Neutralization IC_{50}s evaluated for three mAbs for each epitope specificity and including PGT121 as control. The antibodies were tested for neutralization against BG505 WT as well as K168E, R192K, G354R, and N356K mutant viruses, which were mutations identified by virus sequencing post-challenge in the rhesus macaques. The N168E and R192K mutations are located in the V2 region, and the G354R and N356K mutations are located in the C3 region. Assays were run in duplicate. Error bars represent standard deviation.

(F) nAbs targeting the V1/V3 and C3/V5 epitopes were tested individually and in combination to assess potential neutralization synergies against BG505 pseudovirus. Error bars represent standard deviation.

(G) ADCC activity was measured for nAbs of differing specificities from animal 11M088 using BG505 virus on CEM.CCR5 target cells and macaque NK effector cells. HIV bnAb PGT121 served as positive control and an anti-dengue mAb Den3 as a negative control. Data are representative of two independent experiments. Error bars represent standard deviation.

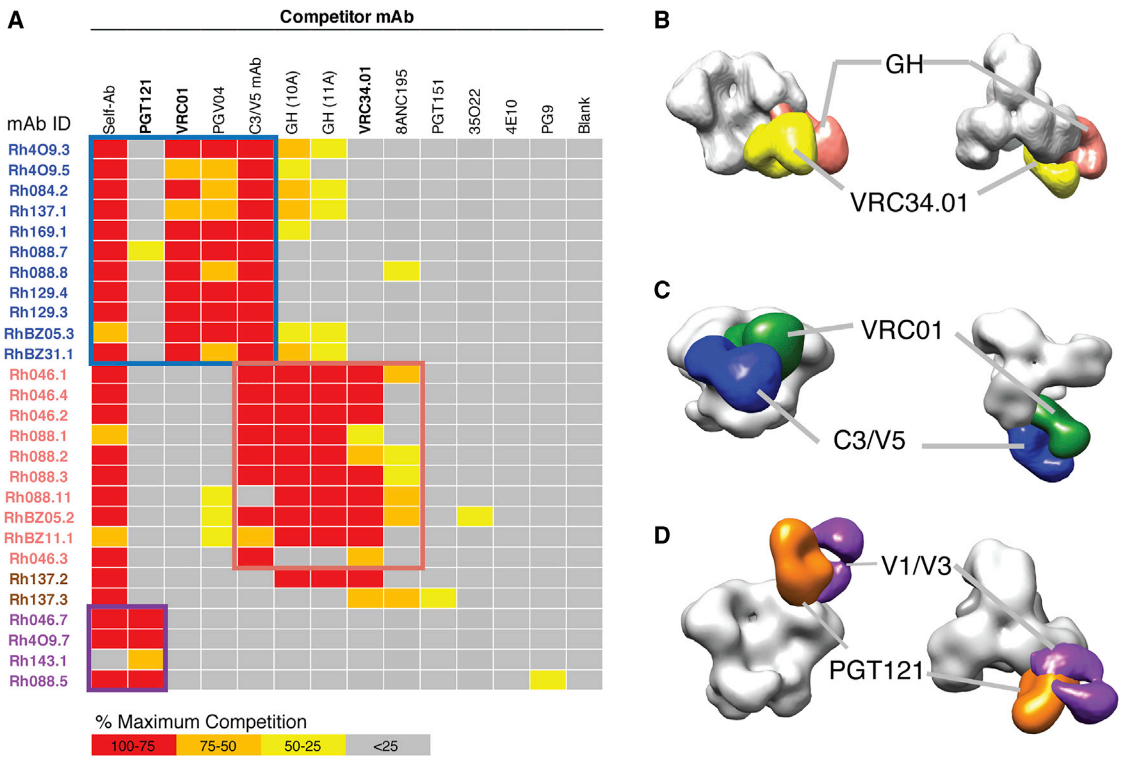

Figure 4. Strain-Specific nAbs Block the Binding of bnAbs to HIV-1 Env

(A) ELISA competition between a panel of HIV-1 bnAbs and rhesus mAbs for binding to BG505 SOSIP. Maximum percentage of competition was colored according to the key. Two rabbit glycan hole (GH)-targeting mAbs (10A,11A). "Self-Ab" indicates competition between the labeled and unlabeled versions of the same rhesus mAb.

(B–D) Negative stain electron microscopy epitope mapping of rhesus mAbs binding to BG505 SOSIP compared to binding by human HIV bnAbs. (B) Overlap of fusion peptide and 241/289 GH epitopes targeted by VRC34.01 (yellow) and RhBZ05.1 (pink). (C) Overlap of CD4bs and C3/V5 epitopes as revealed by the footprints recognized by VRC01 (green) and C3/V5-targeting RhBZ31.1 (blue). (D) Overlap of V3-glycan and V1/V3 epitopes targeted by PGT121 (orange) and Rh046.7 (purple).

antibody-dependent cell-mediated cytotoxicity (ADCC) activity as a synergistic effect. To investigate neutralization synergy, we tested V1/V3 and C3/V5 nAbs individually and in combination for neutralization of BG505 virus, but no evidence of neutralization synergy was observed (Figure 3F). Interestingly, a combination of nAbs to C3/V5 and V1/V3 did show enhanced cell killing that is far greater than the sum of the individual antibodies (Figure 3G). The killing activity of the two antibodies was much greater than PGT121, which was used as a positive control in the assay. It remains to be determined whether such synergistic effector function activity might play a role in protection. We note that the nAbs used for the ADCC assay derive from animal 11M088, which showed delayed infection following virus challenge but was infected nonetheless after 10 challenges. Synergistic ADCC activity was not observed for selected mAbs to V1/V3 and C3/V5 from macaque 4O9 (data not shown).

Diverse BG505 Autologous nAbs Specificities Block HIV-1 bnAbs Binding

We next wanted to determine how the strain-specific nAbs might impact the development of bnAb responses. On the one hand, these responses may have the potential to be affinity-matured to bnAbs, but on the other these responses could distract and reduce the likelihood of developing bnAbs (Bradley et al., 2016). To this end, we measured competition between rhesus mAbs and a panel of human HIV bnAbs by ELISA, and we carried out epitope mapping using EMPEM. The rhesus nAbs to the N241/N289 glycan hole and interface strongly competed with the human gp120-gp41 interface-specific bnAb VRC34.01 (Kong et al., 2016; Figure 4A). They also competed with two glycan-hole rabbit mAbs, 10A and 11A (McCoy et al., 2016). Rh137.2 also strongly competed with bnAb 8ANC195 (Scharf et al., 2015). However, FP-targeting rhesus mAb did not compete with rhesus glycan-hole nAbs (Figure S4A). We further confirmed overlap of the VRC34.01 epitope to the rhesus glycan-hole nAbs by EMPEM (Figure 4B). The nAbs targeting the C3/V5 epitope showed competition with CD4bs-targeting bnAbs including VRC01 (Wu et al., 2010) and PGV04 (Falkowska et al., 2012) due to the close proximity between the CD4bs and the C3/V5 on Env (Figures 4A and 4C). Finally, the nAbs to V1/V3 loop competed strongly with V3-glycan targeting bnAbs, including PGT121 (Walker et al., 2011) and 10-1074 (Mouquet et al., 2012; Figures 4A and S4B). V2-apex targeting bnAbs such as PG9 (Walker et al., 2009) and PGDM1400 (Sok et al., 2014) displayed minimal competition with V1/V3-targeting mAbs, illustrating the angles of approach of these antibodies

are more toward the N137 glycan at V1 loop and possibly toward the V3 loop (Figure 4D; Garces et al., 2014; Sok et al., 2016b). Overall, the four rhesus nAb specificities—N241/N289 glycan hole, V1/V3, C3/V5, and gp120-gp41 interface—all approach their epitopes at different angles from canonical bnAbs bringing into question whether they can be further broadened beyond their strain-specific recognition. None of the rhesus nAbs to C3/V5, V1/V3, interface, or the N241/N289 glycan hole were able to bind any of the global panel of HIV-1 Env trimers (Figure S2C), indicating the difficulty of boosting autologous antibodies with additional trimers. The exception was the FP-targeting mAb Rh137.3, which showed binding to several Env trimers. It remains to be determined whether such responses can be broadened further through subsequent prime/boosts *in vivo*. If these responses cannot be affinity-matured to cross-react to other HIV strains, then the competition data suggest that such responses are likely to occlude binding of HIV bnAb precursors.

Genetic Determinants Might Influence HIV-1 Env Immunogenicity

Unlike rabbits, mice, and cows, the immunodominant epitope in rhesus macaques is the C3/V5 region, followed by the N241/N289 glycan hole, the V1/V3 region, and the gp120-gp41 interface. For the C3/V5 epitope, we noticed that nAbs from three different macaques—one naive infected animal BZ31 and two immunized animals 12-084 and 12-137—targeting this epitope shared very similar clonotypes. We hypothesized that this recombination event might be a "public clonotype" (Setliff et al., 2018) among rhesus macaques that might therefore preferentially elicit nAbs to the C3/V5 epitope region. All 9 mAbs, despite being isolated from three different animals, used the same V_H, D_H, J_H, and V_k segments, same CDRH3 lengths, and closely related CDRH3 amino acid sequences (Figure 5A). On the basis of Ig nucleotide sequences, these antibodies are 89%–96% similar in IgH sequence identity and 92%–98% similar in IgK sequence identity (Figure 5B). On the basis of Ig amino acid sequences, these antibodies shared 82%–95% identity in IgH and 84% to 97% identity in IgK (Figure S5A).

To determine whether this "public clonotype" might outcompete other antibody responses to BG505 SOSIP, we first compared the relative affinities of germline-reverted variants (iGL) for the public clonotype targeting C3/V5 compared to iGLs for antibodies targeting the V1/V3 epitope. The data reveal that the iGL antibodies targeting C3/V5 retain some binding affinity to BG505 SOSIP, whereas the corresponding iGL antibodies for the V1/V3 completely loses binding (Figure 5C). These results suggest that the higher relative affinity of this common VDJ recombination event might confer an advantage over antibodies targeting other epitopes and might therefore favor the immunodominant C3/V5 responses seen in macaques.

We next wanted to determine whether this recombination event can be observed in other rhesus macaques not involved in this study. First, we searched for similar sequences in a previous study that compared immune responses to BG505 SOSIP delivered by osmotic pumps or by bolus injection using the following parameters: sequences containing the same V_H 3-128 gene allele, closely related V_D and V_J gene alleles and 18 amino acid (aa)-CDRH3 length. Interestingly, in Cirelli et al. (2019), a similar clonotype was found in one of the 8 macaques that had matched VDJ genes, CDRH3 length, and a similar "CSSxxCSS" D gene motif in the CDRH3 as the C3/V5-targeting rhesus nAbs in this study (Figure 5D), which suggests that this CDRH3 junction might be relatively common among Indian rhesus macaques. We next searched a naive rhesus macaque next-generation sequencing (NGS) database (Cottrell et al., 2020) for sequences. Strikingly, we found 30 germline HC sequences from 6 naive animals with similar CDRH3s. Many of these sequences also have the "CSSxxCSS" motif in the germline encoded D gene, but the motif is positioned in a different register within the CDRH3 (Figure 5D). To determine whether these naive antibodies might have some neutralizing activity to BG505 despite this difference, we expressed 5 heavy chains identified in the naive germline NGS database paired with the light chain of mAb Rh137.4 to test for neutralization activities against BG505 pseudovirus. Interestingly, one naive heavy chain (GL25) paired with C3/V5 iGL or mature light chain was able to weakly neutralize BG505 virus (Figure 5E). Moreover, the Rh137.1 iGL neutralized BG505 at an IC_{50} of 16 μg/mL. The results confirm that a non-vaccine elicited antibody chimera containing this junction region is able to neutralize BG505 virus.

To determine whether this germline-encoded D gene plays an important role in epitope binding to BG505 SOSIP, we performed alanine-scanning mutagenesis of each residue in the CDRH3 loop from mAb Rh137.4 (Figure 5F). Elimination of the disulfide bond disrupts the function of binding and neutralization activity of the mAb, which indicates that this germline-encoded D gene is indeed important for binding to the C3/V5 epitope (Figure 5F).

To estimate the frequency of the different antibody specificities, we performed B cell receptor (BCR) sequencing of the immunized macaques, including the public clonotype, in the naive and memory repertoire. Given the limited sample availability and therefore limited sampling depth, we first calculated an estimated precursor frequency for each lineage by measuring the combined frequency of V, D, and J genes among the IgM naive repertoire. Interestingly, the public clonotype VDJ recombination frequency appears lower than the VDJs of lineages targeting other epitopes (Figure 5G). Among the IgG memory repertoire, the public clonotype is not detectable in animals BZ31 or 12-137 post-boost or pre-challenge but can be detected in animal 12-084 at <0.15% frequency (Figure S5B). As expected, there is an expansion of this clonotype post-challenge in these animals. The sequencing data also independently confirm the presence of this clonotype across three different macaques and is therefore unlikely to be attributed to contamination (Figure S5C). We do not observe any obvious trends in changes in lineage frequencies for the other specificities over the course of the study, but we do observe higher levels of SHM for antibodies to the glycan hole compared to the V1/V3 and C3/V5 specificities (Figure S5D). Interesting, as mentioned previously, the nAbs to the N241/N289 glycan hole poorly neutralize BG505 despite a relatively high level of SHM. Overall, the data suggest that the "public clonotype" targeting the C3/V5 may not be very common relative to other antibodies in the repertoire, but the recombination event occurs frequently enough to be observed in multiple macaques and likely has an affinity advantage in binding to the C3/

A

mAb ID	Heavy chain V gene allele	Heavy chain D gene allele	Heavy chain J gene allele	SHM (nt)	CDRH3	Light chain V gene allele	Light chain J gene allele	SHM (nt)	CDRL3
RhBZ31.1	VH3-128*01	DH2-10*01	JH5*01	3%	SRDCSSTDCSSSYSRFDV	VK3-7*01	JK1*01	2%	FQYFSGWT
RhBZ31.2	VH3-128*01	DH2-10*01	JH5*01	4%	TRDCSSDFCSSSYNRFNV	VK3-7*01	JK1*01	3%	FQYYSGWT
RhBZ31.3	VH3-128*01	DH2-10*01	JH5*01	4%	TRDCSSTSCSSSYSRFDV	VK3-7*01	JK1*01	4%	FQYFSGWT
Rh084.1	VH3-128*01	DH2-10*01	JH5*01	4%	TRDCSSSSCSWTYSRFDV	VK3-7*01	JK3*01	5%	YQYYSGYT
Rh084.3	VH3-128*01	DH2-10*01	JH5*01	6%	TRDCSSSSCSSRYSRFDV	VK3-7*01	JK2*01	3%	YQYFSGYT
Rh084.2	VH3-128*01	DH2-10*01	JH5*01	6%	TRDCSSSACSSRYSRFDV	VK3-7*01	JK3*01	5%	YQYFSGYT
Rh137.1	VH3-128*01	DH2-10*01	JH5*01	7%	TRDCSSSDCSSLFRRFDV	VK3-7*01	JK2*01	3%	YQYFSGYS
Rh137.4	VH3-128*01	DH2-10*01	JH5*01	7%	TRDCSSSDCSSSFRRFDV	VK3-7*01	JK2*01	7%	YQYFSGYT
Rh137.5	VH3-128*01	DH2-10*01	JH5*01	7%	TRDCSSSDCSSSFRRFDV	VK3-7*01	JK2*01	5%	YQYFSGYT

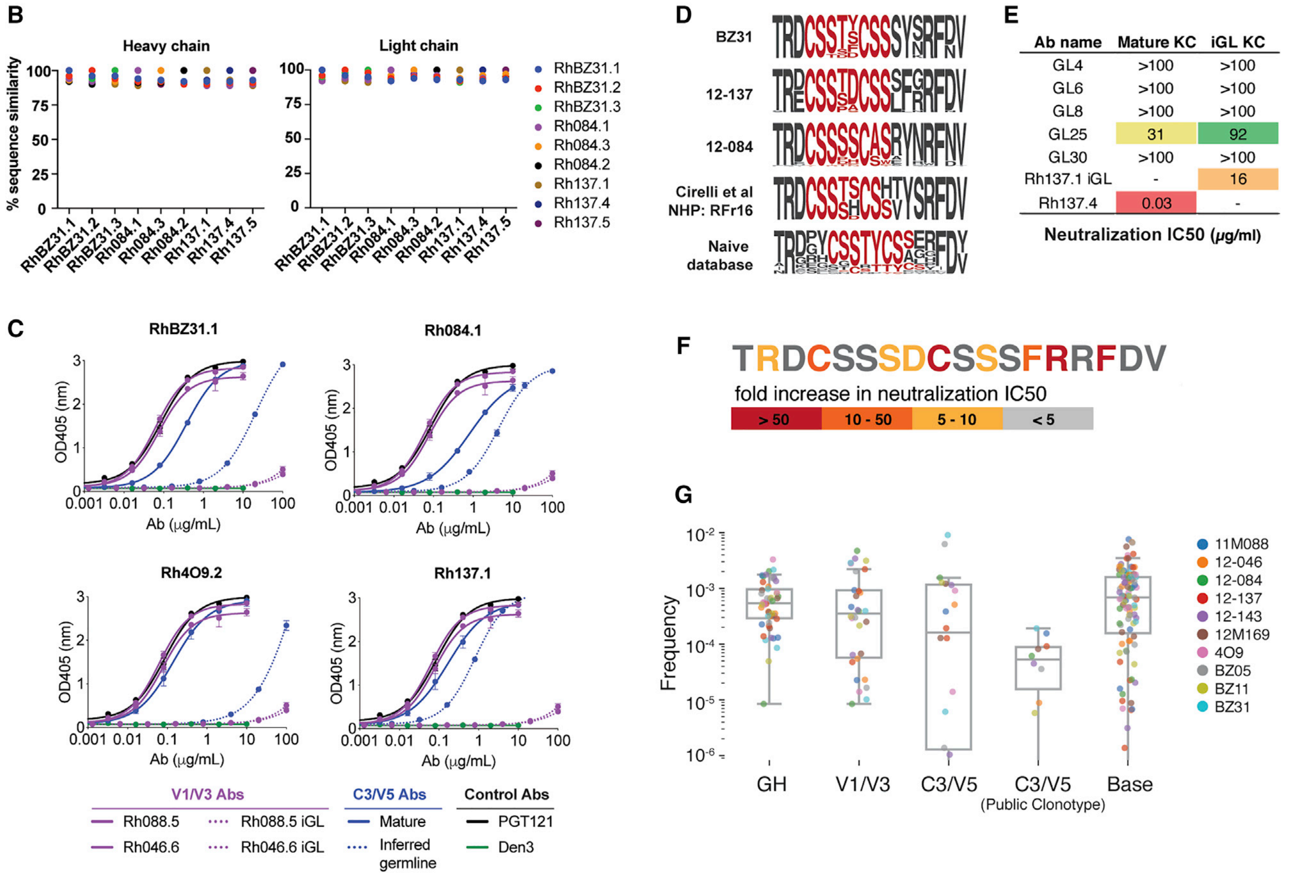

Figure 5. Sequence Characteristics of Closely Related Rhesus mAbs that Recognize the C3/V5 Region of BG505 Env

(A) VDJ genes, CDRH3 sequences, and percentage of SHM of nAbs with shared clonotypes from immunized and infected animals.

(B) Heavy-chain and light-chain nucleotide sequence identities for rhesus mAbs.

(C) Binding ELISA of V1/V3- and C3/V5-targeting nAbs and corresponding inferred germline (iGL) Abs to BG505 SOSIP. Solid line indicates mature nAb, and dashed line indicates iGL mAb. Purple line indicates V1/V3-targeting mAb, while blue line indicates C3/V5-targeting mAb. PGT121 and Den3 are positive and negative controls, respectively. Assays were run in duplicate. Error bars represent standard deviation.

(D) CDRH3 sequence logos for nAbs for macaques BZ31, 12-137, and 12-084 in this study as well as sequences from a previous study (Cirelli et al., 2019) and a macaque database (Cottrell et al., 2020). The D-gene sequence motif is highlighted in red.

(E) Neutralization activity against BG505 pseudovirus of a heavy-chain "public clonotype" sequence from the NGS database paired with either the mature kappa chain (Mature KC) from Rh137.4 or with the inferred germline of Rh137.1 (iGL KC).

(F) Alanine scanning mutations across the CDRH3 for a "public clonotype" antibody to determine which residues are important for neutralization. Values represent the fold increase in neutralization IC_{50} compared to the parental sequence.

(G) Theoretical precursor frequencies based on IgM repertoire V-, D-, J-gene frequency combinations for lineages reported in this study displayed for each rhesus macaque by epitope specificity.

V5 epitope on BG505 SOSIP relative to other antibodies in the naive repertoire.

Finally, we mined antibody sequences in the human antibody repertoire (Briney et al., 2019) to identify CDRH3 junctions that are similar to the rhesus macaque public clonotype antibodies. The closest human D gene that matches the rhesus macaque antibody is IGHD2-2*01, which encodes a "CSSTSC" motif that is similar to the D-gene motif observed in the macaque

nAbs. As shown in Figure 5D, there is sequence variability in this region both in the amino acid composition and also in the position of the D gene that sits within the CDRH3. Given this diversity, it is somewhat difficult to estimate the frequencies of these precursors in the human antibody repertoire, but we provide two approximations. If we calculate the frequency of heavy-chain antibodies with a CDRH3 length of 18 amino acids and an IGDH2-2 gene in any position in the CDRH3 sequence, then the frequency is on average 0.74% (range 0.4%–1.0%) across 10 donors (Figure S6A). If we calculate the frequency of CDRH3 that is most similar to the macaque antibodies, then in 1 of 10 human donors, we identify a sequence that is 5 amino acids different from the closest rhesus nAb (Figure S6B). In 9 of 10 donors, we identify sequences that are 6 amino acids different from the closest rhesus nAbs (Figure S6B). These data indicate that antibodies similar to the public clonotype rhesus macaque antibodies could potentially be elicited in humans following BG505 SOSIP immunization, but this would require experimental confirmation.

DISCUSSION

Neutralizing mAbs to BG505 SOSIP have been reported for rabbits (McCoy et al., 2016), guinea pigs (Lei et al., 2019), and cows (Sok et al., 2017) and few for rhesus macaques (Cottrell et al., 2020). Many anticipate, however, that the antibody responses to BG505 SOSIP in macaques will best mimic the responses anticipated in humans. To this end, we report isolation of 45 nAbs from 9 rhesus macaques, which is the largest dataset to a recombinant HIV trimer from rhesus macaques to date. We anticipate that these nAbs can be used as reagents to help evaluate the specificities of antibody responses to BG505 SOSIP in human clinical trials. We note that these nAbs represent only 2% of the overall antigen-specific memory B cell response, indicating that non-nAbs remain preferentially elicited by recombinant SOSIP immunization, which is likely against the base of the trimer that is inaccessible on virus.

Earlier immunization and SHIV challenge data indicated that a serum neutralization titer of approximately 1:500 is required for protection from infection (Pauthner et al., 2019). The isolation of neutralizing mAbs from these immunized macaques reveals differences in neutralization potency for different epitopes (Figure 2B). The most potent nAbs that neutralize virus to completion are those that target the V1/V3 and C3/V5 epitopes, whereas nAbs to the N241/N289 glycan hole have relatively poor activity with maximum neutralization plateau of only 60% (Figures 2A and 2B). Additionally, because the antibody screening approach is based on neutralization, we did not recover nAbs to the FP, indicating that responses to the FP are mainly binding antibodies. Notably, the antibody binding responses from these same animals were recently described using EMPEM epitope mapping (Nogal et al., 2020) and were highly consistent with isolated nAbs described here. In terms of protection, both C3/V5 and V1/V3 neutralizing antibody specificities may contribute to protection across the 12 repeated low-dose virus challenges. The data also suggest that having two autologous nAb specificities might improve protection efficacy either through increased coverage or through other synergistic or additive effects.

There is an outstanding question as to whether these autologous neutralizing antibody responses can be affinity matured to recognize heterologous viruses or if they might limit the development of bnAbs by diverting immune responses away from conserved epitopes (Derdeyn et al., 2014). Both here and in a recently published study (Nogal et al., 2020), we show that these autologous antibodies target their epitopes at angles-of-approach that differ, sometimes widely, from those of bnAbs, which reduces the likelihood that they could become bnAbs. Moreover, these autologous nAbs also bind to epitopes that are adjacent to bnAb epitopes, which show competition against at least three classes of bnAbs (Figure 4A). We also report the isolation of strain-specific nAbs that can neutralize other viruses but not the BG505 virus, indicating that some weak serum breadth could be due to a combination of strain-specific responses. There remains the possibility that the serum neutralization breadth could be due to a single specificity not isolated in this study. Finally, of the 46 mAbs, we show that all but one of the nAbs fail to bind or neutralize heterologous viruses and would therefore not be selected for in subsequent heterologous boosting experiments. The only exception targets the FP. It remains to be determined whether responses to the FP elicited by trimer immunization can be broadened to neutralize heterologous viruses (Kong et al., 2019). Overall, these data suggest that strain-specific nAbs might occlude access to conserved epitopes and ultimately limit the likelihood of eliciting bnAb responses but that these responses are unlikely to be selected for in a heterologous boosting scheme (Yang et al., 2020).

The isolation of rhesus nAbs in this study also revealed a "public clonotype" (Setliff et al., 2018) that targets the immunodominant C3/V5 epitope. This shared junction region, with shared VDJ for heavy chain and similar VJ for light chain, was found in three different macaques. As the two potent neutralizing specificities are directed to V1/V3 and C3/V5 epitopes, we confirmed that the germline-reverted variants of C3/V5 antibodies have higher binding affinities than the corresponding germline variants for V1/V3 antibodies and propose that this affinity difference likely contributes to the immunodominance of antibodies to the C3/V5 epitope relative to the V1/V3 epitope (Figure 5C). To reduce the likelihood that this observed public clonotype is due to contamination, we confirmed that this public clonotype is present at significant frequency in a public database comprising sequences from other rhesus macaques not involved in the study (Cirelli et al., 2019; Figure 5D). When considering how this finding informs or affects vaccine design, the data suggest that BG505 SOSIP as trimer "polishing" immunogen might preferentially select for this public clonotype instead of elicited bnAb precursors. Vaccine design strategies therefore would need to ensure that elicited bnAb precursors have higher affinities compared to these public clonotypes or, alternatively, to develop an engineered version of the SOSIP with the C3/V5 epitope removed or shielded.

As we wait for data to emerge from the first-in-human trials of BG505 SOSIP.664 in the United States and Kenya (NCT03699241), the data derived from this NHP study already present a few important questions to focus on. First, will humans elicit similar serum nAb responses as macaques and will there be similar patterns of immunodominance to the C3/V5, V1/V3,

gp120-gp41 interface, and glycan hole epitopes? Second, if nAb responses to the C3/V5 epitope are similarly immunodominant in humans, will there also be evidence of public clonotypes in humans to BG505 SOSIP and would the frequencies of these clonotypes differ between the US and African clinical trial sites? Answers to these questions will have important implications for whether rhesus macaques serve as a predictive model for antibody responses in humans and present key considerations for how to introduce a trimer immunogen into a prime/boost sequence to elicit broadly neutralizing antibodies.

STAR★METHODS

Detailed methods are provided in the online version of this paper and include the following:

- KEY RESOURCES TABLE
- RESOURCE AVAILABILITY
  - Lead Contact
  - Materials Availability
  - Data and Code Availability
- EXPERIMENTAL MODELS AND SUBJECT DETAILS
  - Non-Human Primates
  - Cell Lines
- METHOD DETAILS
  - Recombinant Trimer Purification
  - Isolation of BG505 SOSIP.664-specific Memory B cells by Flow Cytometry
  - 384-well Micro-Neutralization Assay
  - RT-PCR, Ig Amplification, and Cloning
  - Antibody Production
  - Env Trimer and gp120 Binding ELISA
  - Competition ELISA
  - Envelope Mutation and Pseudovirus Production
  - TZM-bl Neutralization Assay
  - ADCC Assay
  - CD8 T cell Depletion Study
  - Viral Load Assay
  - Naive Rhesus Macaque Germline Database
  - Next Generation Sequencing
  - Baseline Frequency of Human Antibody Heavy Chains Encoding IGHD2-2 and An 18AA CDRH3
  - Negative-Stain EM
- QUANTIFICATION AND STATISTICAL ANALYSIS

SUPPLEMENTAL INFORMATION

Supplemental Information can be found online at https://doi.org/10.1016/j.celrep.2020.108122.

ACKNOWLEDGMENTS

We thank Colin Havenar-Daughton, Samantha Reiss, and Jeff Lifson for assistance with the CD8 T cell depletion study. We thank Sal Butera for assistance with rhesus sample arrangement. This work was supported by the NIH CHAVD (UM1 AI144462 to A.B.W., B.B., D.R.B., and D.S.) and by the IAVI Neutralizing Antibody Center through the Collaboration for AIDS Vaccine Discovery grant OPP1196345/INV-008813 funded by the Bill and Melinda Gates Foundation (A.B.W., D.R.B., and D.S.). This work was also supported by the John and Mary Tu Foundation and the James B. Pendleton Charitable Trust. This work was funded in part by IAVI and made possible by the support of many donors, including the Bill & Melinda Gates Foundation, the Ministry of Foreign Affairs of Denmark, Irish Aid, the Ministry of Finance of Japan in partnership with The World Bank, the Ministry of Foreign Affairs of the Netherlands, the Norwegian Agency for Development Cooperation (NORAD), the United Kingdom Department for International Development (DFID), and the United States Agency for International Development (USAID). The full list of IAVI donors is available at https://www.iavi.org/. The contents of this manuscript are the responsibility of IAVI and do not necessarily reflect the views of USAID or the US Government.

AUTHOR CONTRIBUTIONS

F.Z., D.R.B., and D.S. conceived and designed the study. F.Z., A.B., A.R., T.B., H.Q., and E.L. performed sorting and antibody sequence experiments. F.Z. and A.B. characterized monoclonal antibodies in functional assays. C.J. and B.B. performed antibody repertoire experiments. B.N., C.A.C., and A.B.W. performed structural studies. R.M. provided protocols for rhesus memory B cell activation. R.N. performed ADCC assays. F.Z., C.J., B.N., R.N., E.L., and B.B. designed experiments and/or analyzed the data. F.Z., D.R.B., and D.S. wrote the manuscript, and all authors reviewed and edited the manuscript.

DECLARATION OF INTERESTS

The authors declare no competing interests.

Received: April 14, 2020
Revised: June 29, 2020
Accepted: August 18, 2020
Published: September 8, 2020

REFERENCES

Alpert, M.D., Heyer, L.N., Williams, D.E.J., Harvey, J.D., Greenough, T., Allhorn, M., and Evans, D.T. (2012). A novel assay for antibody-dependent cell-mediated cytotoxicity against HIV-1- or SIV-infected cells reveals incomplete overlap with antibodies measured by neutralization and binding assays. J. Virol. *86*, 12039–12052.

Andrabi, R., Bhiman, J.N., and Burton, D.R. (2018). Strategies for a multi-stage neutralizing antibody-based HIV vaccine. Curr. Opin. Immunol. *53*, 143–151.

Bianchi, M., Turner, H.L., Nogal, B., Cottrell, C.A., Oyen, D., Pauthner, M., Bastidas, R., Nedellec, R., McCoy, L.E., Wilson, I.A., et al. (2018). Electron-Microscopy-Based Epitope Mapping Defines Specificities of Polyclonal Antibodies Elicited during HIV-1 BG505 Envelope Trimer Immunization. Immunity *49*, 288–300.

Bradley, T., Fera, D., Bhiman, J., Eslamizar, L., Lu, X., Anasti, K., Zhang, R., Sutherland, L.L., Scearce, R.M., Bowman, C.M., et al. (2016). Structural Constraints of Vaccine-Induced Tier-2 Autologous HIV Neutralizing Antibodies Targeting the Receptor-Binding Site. Cell Rep. *14*, 43–54.

Briney, B., Sok, D., Jardine, J.G., Kulp, D.W., Skog, P., Menis, S., Jacak, R., Kalyuzhniy, O., de Val, N., Sesterhenn, F., et al. (2016). Tailored Immunogens Direct Affinity Maturation toward HIV Neutralizing Antibodies. Cell *166*, 1459–1470.

Briney, B., Inderbitzin, A., Joyce, C., and Burton, D.R. (2019). Commonality despite exceptional diversity in the baseline human antibody repertoire. Nature *566*, 393–397.

Burton, D.R. (2019). Advancing an HIV vaccine; advancing vaccinology. Nat. Rev. Immunol. *19*, 77–78.

Cirelli, K.M., Carnathan, D.G., Nogal, B., Martin, J.T., Rodriguez, O.L., Upadhyay, A.A., Enemuo, C.A., Gebru, E.H., Choe, Y., Viviano, F., et al. (2019). Slow delivery immunization enhances HIV neutralizing antibody and germinal center responses via modulation of immunodominance. Cell *177*, 1153–1171.

Cline, A.N., Bess, J.W., Piatak, M., Jr., and Lifson, J.D. (2005). Highly sensitive SIV plasma viral load assay: practical considerations, realistic performance

expectations, and application to reverse engineering of vaccines for AIDS. J. Med. Primatol. 34, 303–312.

Cottrell, C.A., van Schooten, J., Bowman, C.A., Yuan, M., Oyen, D., Shin, M., Morpurgo, R., van der Woude, P., van Breemen, M., Torres, J.L., et al. (2020). Mapping the immunogenic landscape of near-native HIV-1 envelope trimers in non-human primates. bioRxiv. https://doi.org/10.1101/2020.02.05.936096.

de Taeye, S.W., Moore, J.P., and Sanders, R.W. (2016). HIV-1 Envelope Trimer Design and Immunization Strategies To Induce Broadly Neutralizing Antibodies. Trends Immunol. 37, 221–232.

deCamp, A., Hraber, P., Bailer, R.T., Seaman, M.S., Ochsenbauer, C., Kappes, J., Gottardo, R., Edlefsen, P., Self, S., Tang, H., et al. (2014). Global panel of HIV-1 Env reference strains for standardized assessments of vaccine-elicited neutralizing antibodies. J. Virol. 88, 2489–2507.

Derdeyn, C.A., Moore, P.L., and Morris, L. (2014). Development of broadly neutralizing antibodies from autologous neutralizing antibody responses in HIV infection. Curr. Opin. HIV AIDS 9, 210–216.

Doria-Rose, N.A., Schramm, C.A., Gorman, J., Moore, P.L., Bhiman, J.N., DeKosky, B.J., Ernandes, M.J., Georgiev, I.S., Kim, H.J., Pancera, M., et al.; NISC Comparative Sequencing Program (2014). Developmental pathway for potent V1V2-directed HIV-neutralizing antibodies. Nature 509, 55–62.

Falkowska, E., Ramos, A., Feng, Y., Zhou, T., Moquin, S., Walker, L.M., Wu, X., Seaman, M.S., Wrin, T., Kwong, P.D., et al. (2012). PGV04, an HIV-1 gp120 CD4 binding site antibody, is broad and potent in neutralization but does not induce conformational changes characteristic of CD4. J. Virol. 86, 4394–4403.

Feng, Y., Tran, K., Bale, S., Kumar, S., Guenaga, J., Wilson, R., de Val, N., Arendt, H., DeStefano, J., Ward, A.B., and Wyatt, R.T. (2016). Thermostability of Well-Ordered HIV Spikes Correlates with the Elicitation of Autologous Tier 2 Neutralizing Antibodies. PLoS Pathog. 12, e1005767.

Garces, F., Sok, D., Kong, L., McBride, R., Kim, H.J., Saye-Francisco, K.F., Julien, J.-P., Hua, Y., Cupo, A., Moore, J.P., et al. (2014). Structural evolution of glycan recognition by a family of potent HIV antibodies. Cell 159, 69–79.

Gorman, J., Mason, R.D., Nettey, L., Cavett, N., Chuang, G.-Y., Peng, D., Tsybovsky, Y., Verardi, R., Nguyen, R., Ambrozak, D., et al. (2019). Isolation and Structure of an Antibody that Fully Neutralizes Isolate SIVmac239 Reveals Functional Similarity of SIV and HIV Glycan Shields. Immunity 51, 724–734.

Havenar-Daughton, C., Carnathan, D.G., Torrents de la Peña, A., Pauthner, M., Briney, B., Reiss, S.M., Wood, J.S., Kaushik, K., van Gils, M.J., Rosales, S.L., et al. (2016). Direct probing of germinal center responses reveals immunological features and bottlenecks for neutralizing antibody responses to HIV env trimer. Cell Rep. 17, 2195–2209.

Havenar-Daughton, C., Lee, J.H., and Crotty, S. (2017). Tfh cells and HIV bnAbs, an immunodominance model of the HIV neutralizing antibody generation problem. Immunol. Rev. 275, 49–61.

Hu, J.K., Crampton, J.C., Cupo, A., Ketas, T., van Gils, M.J., Sliepen, K., de Taeye, S.W., Sok, D., Ozorowski, G., Deresa, I., et al. (2015). Murine Antibody Responses to Cleaved Soluble HIV-1 Envelope Trimers Are Highly Restricted in Specificity. J. Virol. 89, 10383–10398.

Huang, J., Doria-Rose, N.A., Longo, N.S., Laub, L., Lin, C.-L., Turk, E., Kang, B.H., Migueles, S.A., Bailer, R.T., Mascola, J.R., and Connors, M. (2013). Isolation of human monoclonal antibodies from peripheral blood B cells. Nat. Protoc. 8, 1907–1915.

Jardine, J.G., Ota, T., Sok, D., Pauthner, M., Kulp, D.W., Kalyuzhniy, O., Skog, P.D., Thinnes, T.C., Bhullar, D., Briney, B., et al. (2015). HIV-1 VACCINES. Priming a broadly neutralizing antibody response to HIV-1 using a germline-targeting immunogen. Science 349, 156–161.

Jardine, J.G., Kulp, D.W., Havenar-Daughton, C., Sarkar, A., Briney, B., Sok, D., Sesterhenn, F., Ereño-Orbea, J., Kalyuzhniy, O., Deresa, I., et al. (2016). HIV-1 broadly neutralizing antibody precursor B cells revealed by germline-targeting immunogen. Science 351, 1458–1463.

Joyce, C., Burton, D.R., and Briney, B. (2020). Comparisons of the antibody repertoires of a humanized rodent and humans by high throughput sequencing. Sci. Rep. 10, 1120.

Klasse, P.J., Ketas, T.J., Cottrell, C.A., Ozorowski, G., Debnath, G., Camara, D., Francomano, E., Pugach, P., Ringe, R.P., LaBranche, C.C., et al. (2018). Epitopes for neutralizing antibodies induced by HIV-1 envelope glycoprotein BG505 SOSIP trimers in rabbits and macaques. PLoS Pathog. 14, e1006913.

Kong, R., Xu, K., Zhou, T., Acharya, P., Lemmin, T., Liu, K., Ozorowski, G., Soto, C., Taft, J.D., Bailer, R.T., et al. (2016). Fusion peptide of HIV-1 as a site of vulnerability to neutralizing antibody. Science 352, 828–833.

Kong, R., Duan, H., Sheng, Z., Xu, K., Acharya, P., Chen, X., Cheng, C., Dingens, A.S., Gorman, J., Sastry, M., et al.; NISC Comparative Sequencing Program (2019). Antibody Lineages with Vaccine-Induced Antigen-Binding Hotspots Develop Broad HIV Neutralization. Cell 178, 567–584.

Lander, G.C., Stagg, S.M., Voss, N.R., Cheng, A., Fellmann, D., Pulokas, J., Yoshioka, C., Irving, C., Mulder, A., Lau, P.-W., et al. (2009). Appion: an integrated, database-driven pipeline to facilitate EM image processing. J. Struct. Biol. 166, 95–102.

Lee, J.H., Andrabi, R., Su, C.-Y., Yasmeen, A., Julien, J.-P., Kong, L., Wu, N.C., McBride, R., Sok, D., Pauthner, M., et al. (2017). A Broadly Neutralizing Antibody Targets the Dynamic HIV Envelope Trimer Apex via a Long, Rigidified, and Anionic β-Hairpin Structure. Immunity 46, 690–702.

Lei, L., Yang, Y.R., Tran, K., Wang, Y., Chiang, C.-I., Ozorowski, G., Xiao, Y., Ward, A.B., Wyatt, R.T., and Li, Y. (2019). The HIV-1 Envelope Glycoprotein C3/V4 Region Defines a Prevalent Neutralization Epitope following Immunization. Cell Rep. 27, 586–598.

Link, J.M., Larson, J.E., and Schroeder, H.W. (2005). Despite extensive similarity in germline DH and JH sequence, the adult Rhesus macaque CDR-H3 repertoire differs from human. Mol. Immunol. 42, 943–955.

McCoy, L.E., van Gils, M.J., Ozorowski, G., Messmer, T., Briney, B., Voss, J.E., Kulp, D.W., Macauley, M.S., Sok, D., Pauthner, M., et al. (2016). Holes in the Glycan Shield of the Native HIV Envelope Are a Target of Trimer-Elicited Neutralizing Antibodies. Cell Rep. 16, 2327–2338.

Mouquet, H., Scharf, L., Euler, Z., Liu, Y., Eden, C., Scheid, J.F., Halper-Stromberg, A., Gnanapragasam, P.N.P., Spencer, D.I.R., Seaman, M.S., et al. (2012). Complex-type N-glycan recognition by potent broadly neutralizing HIV antibodies. Proc. Natl. Acad. Sci. USA 109, E3268–E3277.

Nogal, B., Bianchi, M., Cottrell, C.A., Kirchdoerfer, R.N., Sewall, L.M., Turner, H.L., Zhao, F., Sok, D., Burton, D.R., Hangartner, L., and Ward, A.B. (2020). Mapping Polyclonal Antibody Responses in Non-human Primates Vaccinated with HIV Env Trimer Subunit Vaccines. Cell Rep. 30, 3755–3765.

Pauthner, M., Havenar-Daughton, C., Sok, D., Nkolola, J.P., Bastidas, R., Boopathy, A.V., Carnathan, D.G., Chandrashekar, A., Cirelli, K.M., Cottrell, C.A., et al. (2017). Elicitation of robust tier 2 neutralizing antibody responses in nonhuman primates by HIV envelope trimer immunization using optimized approaches. Immunity 46, 1073–1088.

Pauthner, M.G., Nkolola, J.P., Havenar-Daughton, C., Murrell, B., Reiss, S.M., Bastidas, R., Prévost, J., Nedellec, R., von Bredow, B., Abbink, P., et al. (2019). Vaccine-Induced Protection from Homologous Tier 2 SHIV Challenge in Nonhuman Primates Depends on Serum-Neutralizing Antibody Titers. Immunity 50, 241–252.

Ralph, D.K., and Matsen, F.A., 4th. (2016a). Likelihood-Based Inference of B Cell Clonal Families. PLoS Comput. Biol. 12, e1005086.

Ralph, D.K., and Matsen, F.A., 4th. (2016b). Consistency of VDJ rearrangement and substitution parameters enables accurate B cell receptor sequence annotation. PLoS Comput. Biol. 12, e1004409.

Sanders, R.W., and Moore, J.P. (2017). Native-like Env trimers as a platform for HIV-1 vaccine design. Immunol. Rev. 275, 161–182.

Sanders, R.W., Derking, R., Cupo, A., Julien, J.-P., Yasmeen, A., de Val, N., Kim, H.J., Blattner, C., de la Peña, A.T., Korzun, J., et al. (2013). A next-generation cleaved, soluble HIV-1 Env trimer, BG505 SOSIP.664 gp140, expresses multiple epitopes for broadly neutralizing but not non-neutralizing antibodies. PLoS Pathog. 9, e1003618.

Sanders, R.W., van Gils, M.J., Derking, R., Sok, D., Ketas, T.J., Burger, J.A., Ozorowski, G., Cupo, A., Simonich, C., Goo, L., et al. (2015). HIV-1 VACCINES.

CellPress
OPEN ACCESS

HIV-1 neutralizing antibodies induced by native-like envelope trimers. Science *349*, aac4223.

Scharf, L., Wang, H., Gao, H., Chen, S., McDowall, A.W., and Bjorkman, P.J. (2015). Broadly Neutralizing Antibody 8ANC195 Recognizes Closed and Open States of HIV-1 Env. Cell *162*, 1379–1390.

Scheres, S.H.W. (2012). RELION: implementation of a Bayesian approach to cryo-EM structure determination. J. Struct. Biol. *180*, 519–530.

Seaman, M.S., Janes, H., Hawkins, N., Grandpre, L.E., Devoy, C., Giri, A., Coffey, R.T., Harris, L., Wood, B., Daniels, M.G., et al. (2010). Tiered categorization of a diverse panel of HIV-1 Env pseudoviruses for assessment of neutralizing antibodies. J. Virol. *84*, 1439–1452.

Setliff, I., McDonnell, W.J., Raju, N., Bombardi, R.G., Murji, A.A., Scheepers, C., Ziki, R., Mynhardt, C., Shepherd, B.E., Mamchak, A.A., et al. (2018). Multi-Donor Longitudinal Antibody Repertoire Sequencing Reveals the Existence of Public Antibody Clonotypes in HIV-1 Infection. Cell Host Microbe *23*, 845–854.

Sok, D., Laserson, U., Laserson, J., Liu, Y., Vigneault, F., Julien, J.-P., Briney, B., Ramos, A., Saye, K.F., Le, K., et al. (2013). The effects of somatic hypermutation on neutralization and binding in the PGT121 family of broadly neutralizing HIV antibodies. PLoS Pathog. *9*, e1003754.

Sok, D., van Gils, M.J., Pauthner, M., Julien, J.-P., Saye-Francisco, K.L., Hsueh, J., Briney, B., Lee, J.H., Le, K.M., Lee, P.S., et al. (2014). Recombinant HIV envelope trimer selects for quaternary-dependent antibodies targeting the trimer apex. Proc. Natl. Acad. Sci. USA *111*, 17624–17629.

Sok, D., Briney, B., Jardine, J.G., Kulp, D.W., Menis, S., Pauthner, M., Wood, A., Lee, E.-C., Le, K.M., Jones, M., et al. (2016a). Priming HIV-1 broadly neutralizing antibody precursors in human Ig loci transgenic mice. Science *353*, 1557–1560.

Sok, D., Pauthner, M., Briney, B., Lee, J.H., Saye-Francisco, K.L., Hsueh, J., Ramos, A., Le, K.M., Jones, M., Jardine, J.G., et al. (2016b). A Prominent Site of Antibody Vulnerability on HIV Envelope Incorporates a Motif Associated with CCR5 Binding and Its Camouflaging Glycans. Immunity *45*, 31–45.

Sok, D., Le, K.M., Vadnais, M., Saye-Francisco, K.L., Jardine, J.G., Torres, J.L., Berndsen, Z.T., Kong, L., Stanfield, R., Ruiz, J., et al. (2017). Rapid elicitation of broadly neutralizing antibodies to HIV by immunization in cows. Nature *548*, 108–111.

Suloway, C., Pulokas, J., Fellmann, D., Cheng, A., Guerra, F., Quispe, J., Stagg, S., Potter, C.S., and Carragher, B. (2005). Automated molecular microscopy: the new Leginon system. J. Struct. Biol. *151*, 41–60.

Sundling, C., Li, Y., Huynh, N., Poulsen, C., Wilson, R., O'Dell, S., Feng, Y., Mascola, J.R., Wyatt, R.T., and Karlsson Hedestam, G.B. (2012). High-resolution definition of vaccine-elicited B cell responses against the HIV primary receptor binding site. Sci. Transl. Med. *4*, 142ra96.

Tiller, T., Meffre, E., Yurasov, S., Tsuiji, M., Nussenzweig, M.C., and Wardemann, H. (2008). Efficient generation of monoclonal antibodies from single human B cells by single cell RT-PCR and expression vector cloning. J. Immunol. Methods *329*, 112–124.

Walker, L.M., Phogat, S.K., Chan-Hui, P.-Y., Wagner, D., Phung, P., Goss, J.L., Wrin, T., Simek, M.D., Fling, S., Mitcham, J.L., et al.; Protocol G Principal Investigators (2009). Broad and potent neutralizing antibodies from an African donor reveal a new HIV-1 vaccine target. Science *326*, 285–289.

Walker, L.M., Huber, M., Doores, K.J., Falkowska, E., Pejchal, R., Julien, J.-P., Wang, S.-K., Ramos, A., Chan-Hui, P.-Y., Moyle, M., et al.; Protocol G Principal Investigators (2011). Broad neutralization coverage of HIV by multiple highly potent antibodies. Nature *477*, 466–470.

Wu, X., Yang, Z.-Y., Li, Y., Hogerkorp, C.-M., Schief, W.R., Seaman, M.S., Zhou, T., Schmidt, S.D., Wu, L., Xu, L., et al. (2010). Rational design of envelope identifies broadly neutralizing human monoclonal antibodies to HIV-1. Science *329*, 856–861.

Xu, K., Acharya, P., Kong, R., Cheng, C., Chuang, G.-Y., Liu, K., Louder, M.K., O'Dell, S., Rawi, R., Sastry, M., et al. (2018). Epitope-based vaccine design yields fusion peptide-directed antibodies that neutralize diverse strains of HIV-1. Nat. Med. *24*, 857–867.

Yang, Y.R., McCoy, L.E., van Gils, M.J., Andrabi, R., Turner, H.L., Yuan, M., Cottrell, C.A., Ozorowski, G., Voss, J., Pauthner, M., et al. (2020). Autologous Antibody Responses to an HIV Envelope Glycan Hole Are Not Easily Broadened in Rabbits. J. Virol. *94*, e01861–e19.

STAR★METHODS

KEY RESOURCES TABLE

REAGENT or RESOURCE	SOURCE	IDENTIFIER
Antibodies		
APC-Cy7 mouse anti-human CD3 (clone SP34-2)	BD Biosciences	Cat#557757
APC-Cy7 mouse anti-human CD4 (clone OKT4)	Biolegend	Cat#317418
APC-Cy7 mouse anti-human CD8 (clone RPA-T8)	BD Biosciences	Cat#557760
APC-Cy7 mouse anti-human CD14 (clone M5E2)	BD Biosciences	Cat#561384
PerCP/Cy5.5 mouse anti-human CD20 (clone 2H7)	Biolegend	Cat#302326
BV421 mouse anti-human IgG (clone G18-145)	BD Biosciences	Cat#562581
PE mouse anti-human IgM (clone MHM-88)	Biolegend	Cat#314508
BV605 mouse anti-human IgM (clone G20-127)	BD Biosciences	Cat#562977
Goat anti monkey IgG (H+L)	BioRad	Cat#AAI42
Alkaline phosphatase-conjugated goat anti-human Fcγ	Jackson ImmunoResearch	Cat#109-055-190
6x His tag monoclonal antibody	Invitrogen	Cat#MA1-21315
Cell-depleting anti-CD8a antibody	Mass Biologics	Cat#M-T807
Rh4O9.1-RhBZ31.3	This paper	N/A
Bacterial and Virus Strains		
12-virus panel - global isolates	(deCamp et al., 2014)	N/A
BG505.W6M.ENV.C2	NIH AIDS Reagent Program	Cat#11518
MG505.W0M.ENV.A2	NIH AIDS Reagent Program	Cat#11528
Chemicals, Peptides, and Recombinant Proteins		
BG505 SOSIP.664	(Sanders et al., 2013)	N/A
398F1 SOSIP.664	(deCamp et al., 2014)	N/A
X-tremeGENE 9	Roche	Cat#06365809001
Protein A Sepharose	GE Healthcare	Cat#17-1279-03
Recombinant human IL-2	Roche	Cat#11147528001
Recombinant human IL-21	Invitrogen	Cat#PHC0215
Recombinant human IL-4	Miltenyi	Cat#130-093-922
Alkaline phosphatase-conjugated streptavidin	Jackson ImmunoResearch	Cat#016-050-084
Critical Commercial Assays		
Pierce Fab preparation kit	ThermoFisher	Cat#44985
TurboCapture 384 mRNA kit	QIAGEN	Cat#72271
Deposited Data		
Rh4O9.1-RhBZ31.3	GenBank	GenBank MT800659 - MT800752
NGS Sequencing Data	NCBI Sequencing Read Archive	BioProject PRJNA647986
Negative-stain map of RhBZ05.1 Fab in complex with BG505 SOSIP.664	EMDataBank	EMD-22372
Negative-stain map of RhBZ31.1 Fab in complex with BG505 SOSIP.664	EMDataBank	EMD-22373

(*Continued on next page*)

Continued

REAGENT or RESOURCE	SOURCE	IDENTIFIER
Negative-stain map of RhBZ31.2 Fab in complex with BG505 SOSIP.664	EMDataBank	EMD-22374
Negative-stain map of Rh137.3 Fab in complex with BG505 SOSIP.664	EMDataBank	EMD-22385
Negative-stain map of Rh046.7. Fab in complex with BG505 SOSIP.664 (1 Fab bound)	EMDataBank	EMD-22387
Negative-stain map of Rh046.7. Fab in complex with BG505 SOSIP.664 (2 Fabs bound)	EMDataBank	EMD-22388
Experimental Models: Cell Lines		
TZM-bl cells	NIH AIDS Reagent Program	Cat#8129
HEK293T cells	ATCC	Cat#CRL-3216
FreeStyle HEK293F cells	ThermoFisher	Cat#R79007
3T3msCD40L cells	(Huang et al., 2013)	N/A
CEM.NKR-CCR5-sLTR-Luc Cells	(Alpert et al., 2012)	N/A
Experimental Models: Organisms/Strains		
Indian-origin rhesus macaque	(Pauthner et al., 2017)/AlphaGenesis Inc	N/A
Recombinant DNA		
pSG3Δenv plasmid	NIH AIDS Reagent Program	Cat#11051
Software and Algorithms		
FlowJo v9.9.4	FlowJo	https://www.flowjo.com
Prism v7.0	GraphPad	https://www.graphpad.com/scientific-software/prism/
Appion database	(Lander et al., 2009)	N/A
Leginon	(Suloway et al., 2005)	N/A
Relion	(Scheres 2012)	N/A

RESOURCE AVAILABILITY

Lead Contact
Further information and requests for resources and reagents should be directed to and will be fulfilled by the Lead Contact, Devin Sok (dsok@iavi.org).

Materials Availability

- Antibody plasmids in this study are available with a material transfer agreement (MTA)
- Pseudovirus mutants in this study are available with a material transfer agreement (MTA)

Data and Code Availability

- Antibody sequences are available on GenBank (MT800659 - MT800752)
- NGS sequencing data available on NCBI sequencing read archive (BioProject PRJNA647986)
- EM structures deposited to EMDataBank (EMD-22372 - EMD-22385, EMD-22387 - EMD-22388)

EXPERIMENTAL MODELS AND SUBJECT DETAILS

Non-Human Primates
Non-human primates were immunized, SHIV challenged and became infected as described in Pauthner et al., 2019 (Pauthner et al., 2019). Rhesus macaque designations are as given in Pauthner et al., 2019. The sexes of animals as reported in Pauthner et al., 2017 and Pauthner et al., 2019 are as follows: 5 Females (12-137, 12-143, 12M169, 11M088, 4O9), 4 Males (12-046, BZ05, BZ31, BZ11). All rhesus macaques in the immunization study were 3-4 years old at the time of first immunization. In the follow up challenge study, all

macaques were 4-5 years old. The Scripps Research Institutional Animal Care and Use Committee (IACUC) approved all experimental procedures involving all the animals.

Cell Lines

Irradiated 3T3msCD40L cells were used in single B cell culture assay. TZM-bl cells (NIH AIDS Reagents Program) were used in pseudovirus neutralization assay. Human HEK293T cells (ATCC) were used for pseudovirus production. FreeStyle HEK293 cells (ThermoFisher) was used for recombinant trimer production and monoclonal antibody production, CEM.NKR-CCR5-sLTR-Luc cells were used in ADCC assay.

METHOD DETAILS

Recombinant Trimer Purification

BG505 SOSIP.664-Avi gp140, BG505 SOSIP.664-His and other HIV Env trimers were expressed in FreeStyle 293F cells (ThermoFisher) as described previously (Walker et al., 2011). Supernatants were purified by affinity chromatography using a PGT145 column. Trimer with AviTags were biotinylated *in vitro* using the BirA enzyme (Avidity) according to the manufacturer's instructions. The affinity-purified trimers were purified by size exclusion using a HiLoad 26/600 Superdex 200 pg column as described previously (Lee et al., 2017).

Isolation of BG505 SOSIP.664-specific Memory B cells by Flow Cytometry

Cryopreserved PBMCs were thawed, washed, and stained with an antibody cocktail of CD3 (clone SP34-2, BD Biosciences), CD4(clone OKT4, Biolegend), CD8 (clone RPA-T8, BD Biosciences), CD14 (clone M5E2, BD Biosciences), CD20 (clone 2H7, Biolegend), IgM (clone G20-127, BD Biosciences or clone MHM-88, Biolegend), IgG (clone G18-145, BD Biosciences) and fluorescently labeled BG505 Env at room temperature for 20 min in the dark. BG505 SOSIP.664 was labeled with two fluorophores separately, from which dual positive Env-specific ($CD3^-CD4^-CD8^-CD14^-CD20^+IgM^-IgG^+$BG505 $SOSIP^{2+}$) and/or 398F1 $SOSIP^+$ memory B cells were analyzed with a FACSFusion sorter and then single-cell sorted, cultured, and expanded as previous described (Huang et al., 2013). In brief, cells were sorted into Iscove's modified Dulbecco's medium (IMDM) with GlutaMAX (GIBCO) supplemented with 10% heat-inactivated fetal bovine serum (FBS), 1X MycoZap Plus-PR (Lonza), 100 U/mL IL-2 (Roche), 50 ng/mL IL-21 (Invitrogen), 50 ng/mL IL-4 (Miltenyi), 0.1 µg/mL anti-rhesus IgG (H+L) (BioRad) and irradiated 3T3msCD40L feeder cells.

384-well Micro-Neutralization Assay

After 13 to 14 days of culture, B cell supernatants were harvested and screened in a micro-neutralization assay as previous described (Huang et al., 2013). In brief, 20 µL of culture supernatants were incubated with 20 µL of pseudovirus at 37°C for 1 h. 20 µL of TZM-bl cells with diluted dextran were added to each well at 150,000 cells/mL and incubated for 48h at 37°C. Then supernatant was removed, TZM-bl cells were then lysed, luciferase activity was measured by adding BrightGlo (Promega) according to manufacturer's instructions. Wells with both IgG ELISA positive and over 50% neutralization activity were considered as neutralization positive.

RT-PCR, Ig Amplification, and Cloning

After removing culture supernatant, 384-well plates were lysed, and RNA was extracted using TurboCapture 384 mRNA kit (QIAGEN). Single cell RNA from neutralization positive wells were reverse transcribed as described previously (Wu et al., 2010). Subsequently, nested PCR reactions of Ig heavy chain and light chain variable regions were performed in 25 µL volume with 2 µL cDNA transcript. For the first round of PCR in 25 µL reaction, 2.5 µL of 10X PCR buffer (QIAGEN), 0.25 µL of HotStarTaq Plus DNA Polymerase (QIAGEN), 0.5 µL of dNTP (10 mM) (ThermoFisher), 0.25 µL of MgCl2 (25 µM), 0.25 µL of forward primer mixture (50 µM each primer), 0.25 µL of reverse primer mixture (25 µM each primer), 19 µL of DEPC-treated water (ThermoFisher), 2 µL of gene transcripts. Rhesus primers and PCR program are described previously (Gorman et al., 2019). 2 µL of PCR1 products were amplified by nested PCR using 5 µL of 5X Phusion HF buffer (ThermoFisher), 0.24 µL of Phusion HF DNA polymerase (2 U/µL, ThermoFisher), 0.5 µL of dNTP, 0.25 µL of forward primers and 0.25 µL of reverse primers with Gibson adaptor sequences, 0.75 µL of 2X MgCl2, using the following PCR program: 30 s at 98C, followed by 35 cycles of 10 s at 98C, 40 s at 72C, and a final extension for 5 min. Amplified PCR products were analyzed with 2% 96 E-gel (Thermofisher). Antibodies with both recovered heavy chains and light chains were cloned into the corresponding Igγ1, Igκ, Igλ expression vectors (Wu et al., 2010).

Antibody Production

Antibody heavy chain and light chain plasmids were cotransfected at a 1:1 ratio with transfection reagent PEI in HEK293F cells. After 4 to 5 days of transfection, cells were harvested and spun down, supernatants were collected after sterile filtration (0.22 µm). Antibody was purified by Protein A Sepharose (GE Healthcare) as previously described (Sok et al., 2013).

Env Trimer and gp120 Binding ELISA

Streptavidin (Jackson ImmunoResearch) was coated at 2 µg/mL onto high-binding 96-well plates (Corning) overnight or at 37°C for 2 h. After washing, plates were blocked with PBS/3% BSA at RT for 1 h. After further washing, biotinylated BG505 SOSIP.664-Avi or 398F1 SOSIP.664-Avi or BG505 gp120-Avi was added at 1 µg/mL for 1 h at RT. After washing, serially diluted antibodies in PBS/1% BSA were

added to wells and incubated at RT for 1 h. Detection was measured with alkaline phosphatase-conjugated goat anti-human IgG Fcγ (Jackson ImmunoResearch) at 1:1000 dilution for 1h. After final wash, phosphatase substrate (Sigma-Aldrich) was added into wells. Absorption was measured at 405 nm. Non-linear regression curves were analyzed using Prism 8 software to calculate EC_{50} values.

Competition ELISA

Competition ELISA was performed as previously described (Sok et al., 2014). In brief, the antibody of interest was biotinylated following manufacture's description (ThermoFisher). 96-well plates were coated with 6X His tag monoclonal antibody (Invitrogen) overnight. After washing, plates were blocked with PBS/3% BSA at RT for 1 h. BG505 SOSIP.664-His was added into wells at 1 μg/mL and incubated for 1 h. After washing, serially diluted competing antibodies in 1% BSA were added into wells and incubated for 30 min at RT. Biotinylated antibody was then added at the concentration of EC_{70} and incubated at RT for 30-60 min. After washing, alkaline phosphatase-conjugated streptavidin (Jackson ImmunoResearch) was added into wells at RT for 1 h. After final wash, phosphatase substrate (Sigma-Aldrich) was added into wells. Absorption was measured at 405 nm. Non-linear regression curves were analyzed using Prism 8 software to calculate EC_{50} values.

Envelope Mutation and Pseudovirus Production

Pseudovirus envelop plasmid mutation was introduced by site-directed mutagenesis with a QuikChange Site-Directed Mutagenesis kit (Aligent). Pseudovirus Env plasmid was cotransfected with *env*-deficient backbone plasmid (pSG3ΔEnv) in a 1:2 ratio with transfection reagent X-tremeGENE 9 (Roche) in HEK293T cells. After 72 h of transfection, pseudoviruses were harvested by sterile filtration (0.22 μm) of cell culture supernatants and stored at −80°C.

TZM-bl Neutralization Assay

Serially diluted serum or mAbs were incubated with pseudovirus 37°C for 1 h, then transferred onto TZM-bl cells in half-area 96-well plates (Corning). After 48-72 h of incubation, supernatant was removed, TZM-bl cells were then lysed, luciferase activity was measured by adding BrightGlo (Promega) according to manufacturer's instructions. Neutralization were tested in duplicate wells. Each data is representative of at least two independent experiments. Neutralization ID_{50} or IC_{50} titers were calculated using "One-Site Fit LogIC_{50}" regression and by constraining through 0% and 100% neutralization in GraphPad Prism 8.0.

ADCC Assay

CEM.NKR-CCR5-sLTR-Luc cells (Alpert et al., 2012), which express luciferase upon infection, were infected with BG505n332. 2 days post infection serial dilutions of antibodies were added to the target cells and incubated for 15 min. For ADCC synergy test, 0.5X of first antibody and 0.5X of second antibody were mixed and compared to 1X single antibody respectively. Primary human NK cells were added for an approximate 10 to 1 effector to target ratio. Cells were incubated overnight. Luciferase activity was measured using BrightGlo (Promega). Uninfected or infected cells incubated with NK cells in the absence of antibody were used to determine background and maximal luciferase activity, respectively. The dose-dependent loss of luciferase activity represents the antibody-dependent killing of productively infected target cells.

CD8 T cell Depletion Study

Rhesus macaques were injected subcutaneously with 50 mg/kg of anti-CD8a (M-T807, Mass Biologics) antibody, to temporarily ablate $CD8^+$ T cells and NK cells. Depletion was verified by flow cytometry stating of rhesus PBMCs. $CD8^+$ T cells were determined by gating on live/$CD20^-$/$CD3^+$/$CD4^-$ single cells as previously described (Pauthner et al., 2019).

Viral Load Assay

Viral loads were determined by the Quantitative Molecular Diagnostics Core as part of the AIDS and Cancer Virus Program at the Frederick National Laboratory at NIH as previously described (Cline et al., 2005). The sensitivity of the assay was 6 SIV gag RNA copy equivalents per mL.

Naive Rhesus Macaque Germline Database

Germline NGS database is available online http://ward.scripps.edu/gld/. To search for a similar C3/V5 germline rearrangement, LJI.Rh_IGHV3.128 was searched with 100% cutoff, while IGHD2-13*01 and IGHJ4*01 were selected with 80% cutoff, with 18 CDRH3 amino acid length.

Next Generation Sequencing

Total RNA from PBMCs was extracted (RNeasy Maxi Kit, QIAGEN) from each rhesus macaque for the following time-points: Naive, Post-Boost, Pre-Challenge and Post Challenge. Antibody sequences were amplified as previously described (Joyce et al., 2020) with the exception of different primers used during reverse transcription (IgM: ACACTCTTTCCCTACACGACGCTCTTCCGATCTNNNNNNNNNNNNGTCGGGAAGGAAGTCCTGTGCGAG, IgG: ACACTCTTTCCCTACACGACGCTCTTCCGATCTNNNNNNNNNNNNCACCTTGGTGTTGCTGGGCTTGT, IgA: ACACTCTTTCCCTACACGACGCTCTTCCGATCTNNNNNNNNNNNNGAGGCTCAGCGGGAAGACCTTG). Correct PCR product sizes were verified on an agarose gel (E-Gel EX; Invitrogen) and quantified with fluorometry

(Qubit; Life Technologies), pooled at approximately equimolar concentrations and each sample pool was re-quantified before sequencing on an Illumina MiSeq (MiSeq Reagent Kit v3, 600-cycle).

The Abstar analysis pipeline was used as previously described (Joyce et al., 2020) to quality trim, remove adapters, and merge paired sequences. Sequences were then annotated with Abstar in combination with UMI based error correction by AbCorrect (https://github.com/briney/abtools/). Resulting annotated consensus sequences were deposited to MongoDB and Spark databases for querying and data analysis in python on Jupyter and Zeppelin Notebooks.

For the clonal family clustering and identification of nAb lineages in NGS data (related to Figure S5C), Partis (Ralph and Matsen, 2016b, 2016a) was used. Specifically, the subcommand "partition" was carried out with the sequence of each nAb utilized as cluster "seeds."

Baseline Frequency of Human Antibody Heavy Chains Encoding IGHD2-2 and An 18AA CDRH3

We used sequencing data from 10 healthy adult subjects (Briney et al., 2019) compute baseline frequencies. To reduce skewing by highly transcriptionally active cells, multiple sequences from the same subject encoding the same V-gene and HCDR3 amino acid sequence were only counted once. For each subject, the number of productive sequences (no ambiguous nucleotides or stop codons in the junctional region) encoding both an 18AA HCDR3 and the germline gene IGHD2-2 were computed and divided by the total number of productive sequences. Database queries were performed in PySpark using an Elastic MapReduce cluster on Amazon Web Services. Raw and annotated sequences used to compute these baseline frequencies can be found at https://www.github.com/briney/grp_paper.

Negative-Stain EM

Monoclonal Fabs were incubated with BG505 SOSIP.664 trimer (0.3 mg/mL) in 6X molar excess overnight in TBS buffer at RT. Samples were diluted approximately 25-fold prior to applying to glow-discharged, carbon-coated 400-mesh copper grids, followed by pipetting 4 uL of 2% (w/v) uranyl formate then immediately blotting the grid dry. Another 3-4 uL of stain was applied to the grid for 30-60 s, followed by blotting. Air-dried grids were stored under RT until ready for imaging. Tecnai T12 electron microscope was used to collect image data at 120 kV and 52,000x magnification. Leginon software was used for image collection (Suloway et al., 2005). Appion software was used to pick particles from raw images, followed by placement of particles into stacks and 2D reference-free alignment via MSA/MRA (Lander et al., 2009). The particle stacks were subsequently converted from IMAGIC to RELION-formatted MRC stacks and 2D/3D classified (Scheres, 2012) .

QUANTIFICATION AND STATISTICAL ANALYSIS

Due to the small animal subject sample sizes and thus insufficient power to detect significant polyclonal response differences among the unimmunized naive, low titer, and high titer animals, no statistical tests were applied for the purpose of describing the trends in responses and their diversity. Where appropriate, standard deviations were calculated between technical replicates using Graphpad Prism 7.0.

Cell Reports, Volume *32*

Supplemental Information

Mapping Neutralizing Antibody Epitope Specificities to an HIV Env Trimer in Immunized and in Infected Rhesus Macaques

Fangzhu Zhao, Collin Joyce, Alison Burns, Bartek Nogal, Christopher A. Cottrell, Alejandra Ramos, Trevor Biddle, Matthias Pauthner, Rebecca Nedellec, Huma Qureshi, Rosemarie Mason, Elise Landais, Bryan Briney, Andrew B. Ward, Dennis R. Burton, and Devin Sok

Supplemental Information

Mapping neutralizing antibody epitope specificities to an HIV Env trimer in immunized and in infected rhesus macaques

Fangzhu Zhao, Collin Joyce, Alison Burns, Bartek Nogal, Christopher A. Cottrell, Alejandra Ramos, Trevor Biddle, Matthias Pauthner, Rebecca Nedellec, Huma Qureshi, Rosemarie Mason, Elise Landais, Bryan Briney, Andrew B. Ward, Dennis R. Burton, Devin Sok

A

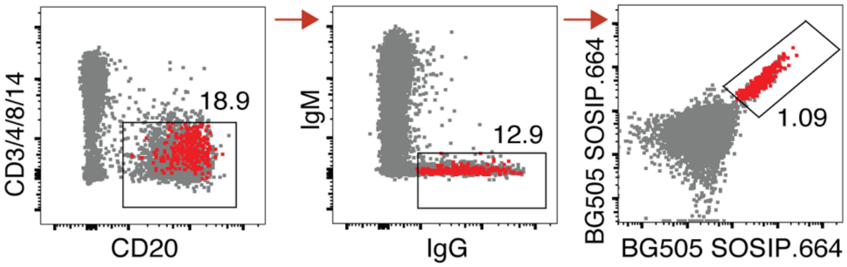

B

Group	Sample	Sorted Cells	IgG+ Wells	Neut+ Wells	Cloned mAbs
IMM	12-084_WK25	524	188	3	3
IMM	12-046_WK26	723	155	4	2
IMM	4O9_WK68	562	80	9	5
IMM	12-137_WK26	768	224	0	0
IMM	12-143_WK26	418	136	0	0
NON-INF	4O9_WK88	76	17	0	0
NI	BZ31_WK86	240	81	5	3
NI	BZ05_WK86	220	32	4	3
IMM	11M088_WK28	720	139	2	1
IMM	12M169_WK28	240	43	0	0
NI	BZ11_WK86	1008	378	1	1
NI	BZ15_WK86	1056	331	0	0
NON-INF	4O9_WK76	484	158	4	3
NON-INF	12-046_WK76	198	44	6	4
INF	11M088_WK86	440	73	11	10
INF	12M169_WK86	176	31	4	3
IMM	12-143_WK26	792	272	1	1
IMM	12M169_WK26	528	159	2	1
INF	12-143_WK70	396	163	1	1
IMM	12-137_WK26	1056	393	5	4
IMM	12-137_WK76	264	98	2	2
	**Total**	**10889**	**3195**	**64**	**47**

Figure S1. FACS sorting and neutralization screening details. Related to Figure 1. (**A**) FACS sorting layout from rhesus PBMCs using biotinylated BG505 SOSIP.664-Avi after gating on lymphocytes/singlets. Events that are BG505 SOSIP.664^{++} are shown in red. (**B**) Summary number of sorted cells, wells with detectable secreted IgG, wells with positive BG505 neutralization, and cloned mAbs from all rhesus PBMCs samples.

A

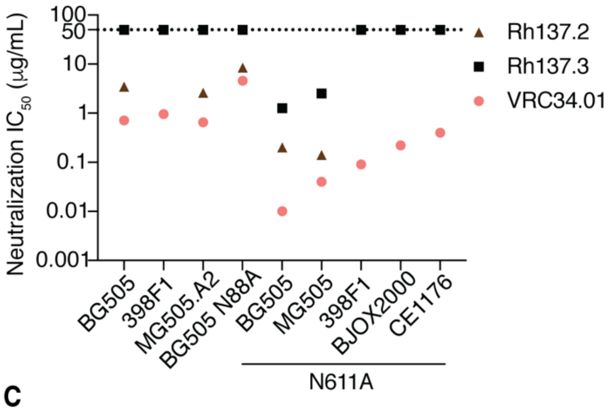

B

	MG505.A2	MG505 CTNN	MG505 CTNA	MG505.A2 Loop H3	MG505.H3 Loop A2
Rh088.5	0.16	>50	6.65	>50	0.01
Rh4O9.7	0.63	>50	6.18	>50	0.05
Rh4O9.8	0.30	>50	12.02	>50	0.04
Rh046.6	0.06	>50	1.12	>50	0.004
Rh046.7	0.31	>50	>50	>50	0.04
Rh143.1	47.38	>100	>100	>100	>100
Rh137.2	2.25	2.07	3.27	3.91	>50
PG9	0.10	0.05	0.03	0.08	0.01
PGT121	0.03	8.06	2.93	>50	>50

C

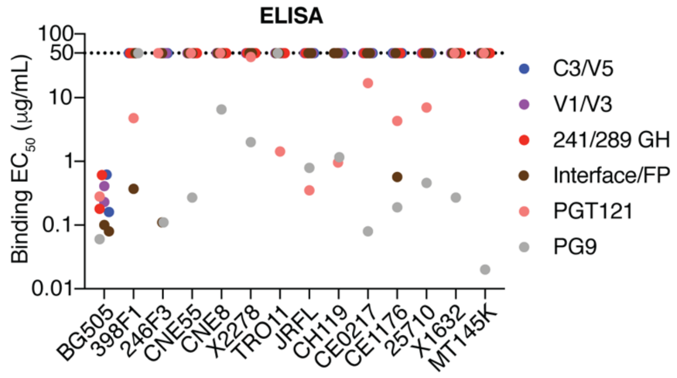

D

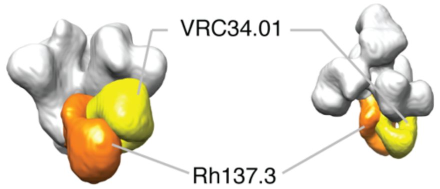

Figure S2. BG505 nAbs remain autologous and do not bind other HIV Env trimers. Related to Figure 2. (**A**) Neutralization IC_{50}s evaluated for gp120-gp41 interface-targeting mAbs Rh137.2, Rh137.3, and including VRC34.01 as control bnAb. Antibodies were tested for neutralization against wildtype BG505, 398F1, MG505.A2 viruses, BG505 N88A mutant, and N611 glycan-deleted virus mutants. (**B**) Neutralization IC_{50}s evaluated for V1/V3-targeting mAbs (purple), gp120-gp41 interface-targeting mAb Rh137.3 (brown) and PGT121 and PG9 as control bnAbs. Antibodies were tested for neutralization against wildtype MG505.A2, MG505.A2 V1 loop mutants including CTNN, CTNA, and CTNNVTNA (Loop H3), and MG505.H3 V1 loop mutant (CTNVTNN). (**C**) ELISA binding EC_{50}s evaluated for two mAbs for each epitope specificity and including PGT121 and PG9 as controls. Antibodies were tested for binding against a set of HIV Env trimers from an indicator panel of global viruses and MT145K as a SIV Env trimer (Andrabi et al., 2019). (**D**) Overlap of fusion peptide epitopes targeted by VRC34.01 (yellow) and Rh137.3 (orange).

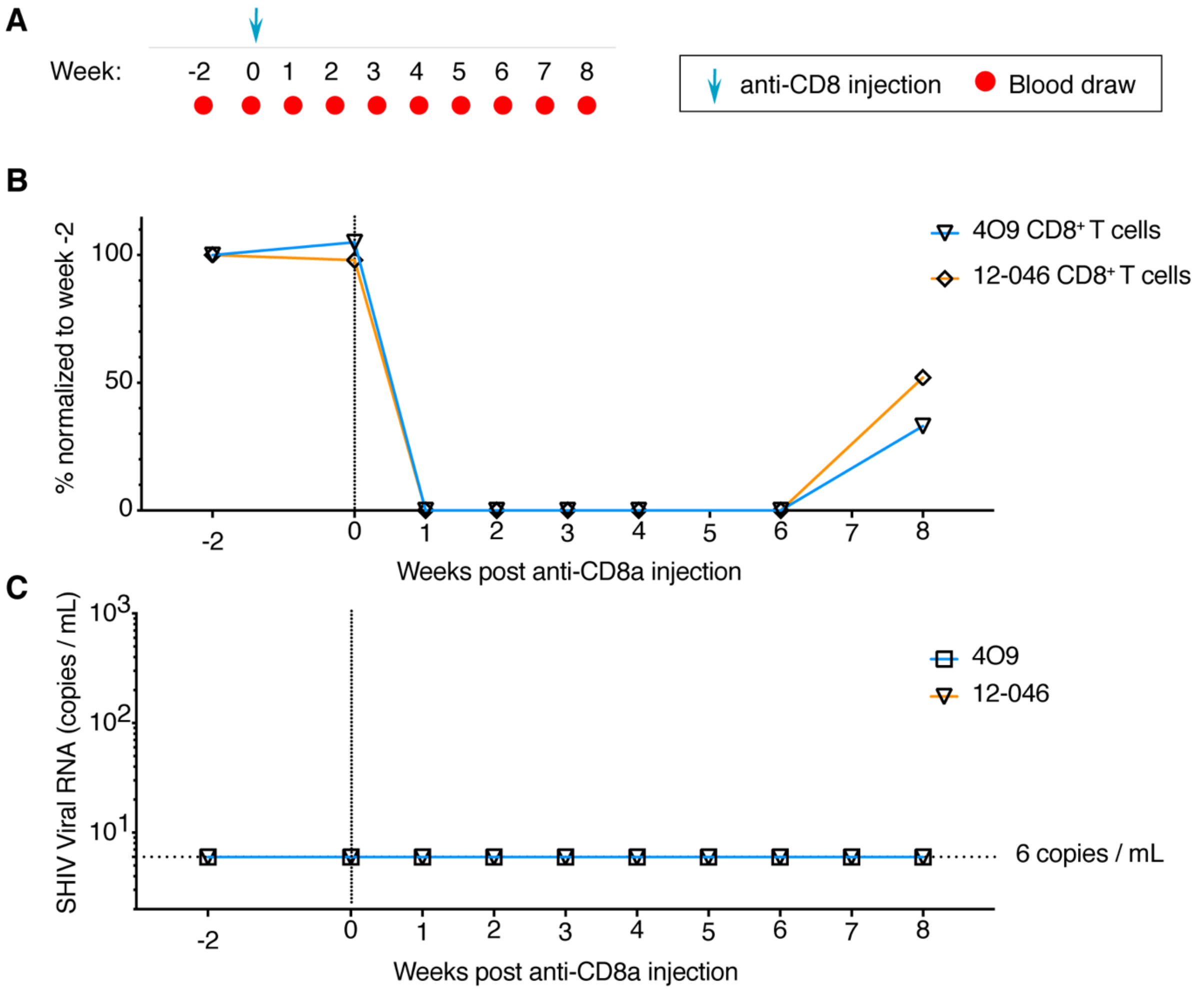

Figure S3. CD8+ T cell depletion does not affect protection against $SHIV_{BG505}$ challenge. Related to Figure 3. (**A**) Animals 12-046 and 4O9 were injected with anti-CD8a antibody after the SHIV challenge study. (**B**) $CD8^+$ T cell frequency after anti-CD8a mAb injection normalized to week -2 in animals 12-046 (orange) and 4O9 (blue). (**C**) SHIV viral load in animals 12-046 (orange) and 4O9 (blue) after anti-CD8a antibody injection.

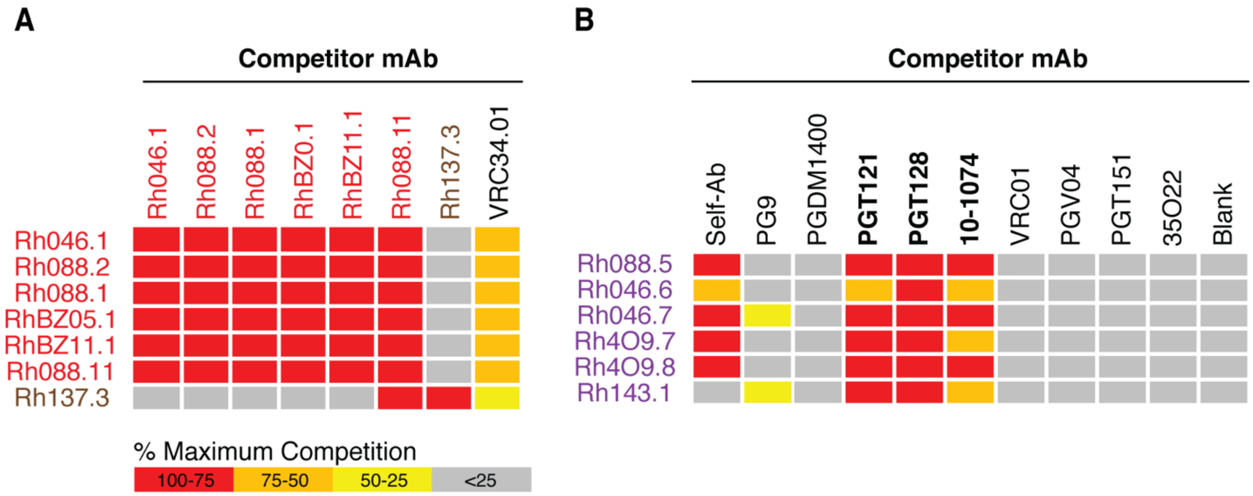

Figure S4. Competition ELISA of strain-specific nAbs and human bnAbs. Related to Figure 4. (A-B) ELISA competition between HIV-1 bnAbs and rhesus mAbs for binding to BG505 SOSIP and colored according to the key. (**A**) Competition between 241/289 GH-targeting mAbs, FP-targeting rhesus mAb Rh137.3, and human bnAb VRC34.01. (**B**) Competition between V1/V3-targeting mAbs and human HIV bnAbs. "Self-Ab" indicates competition between the labelled and unlabeled versions of the same rhesus mAb.

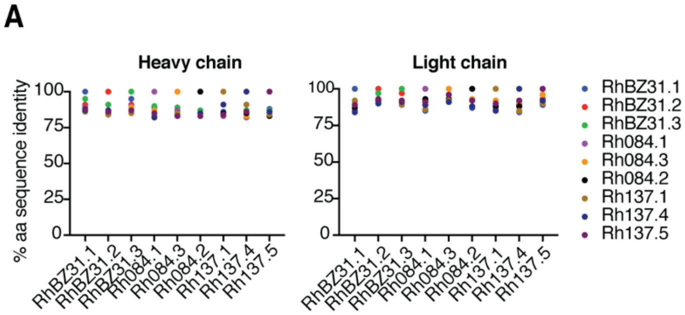

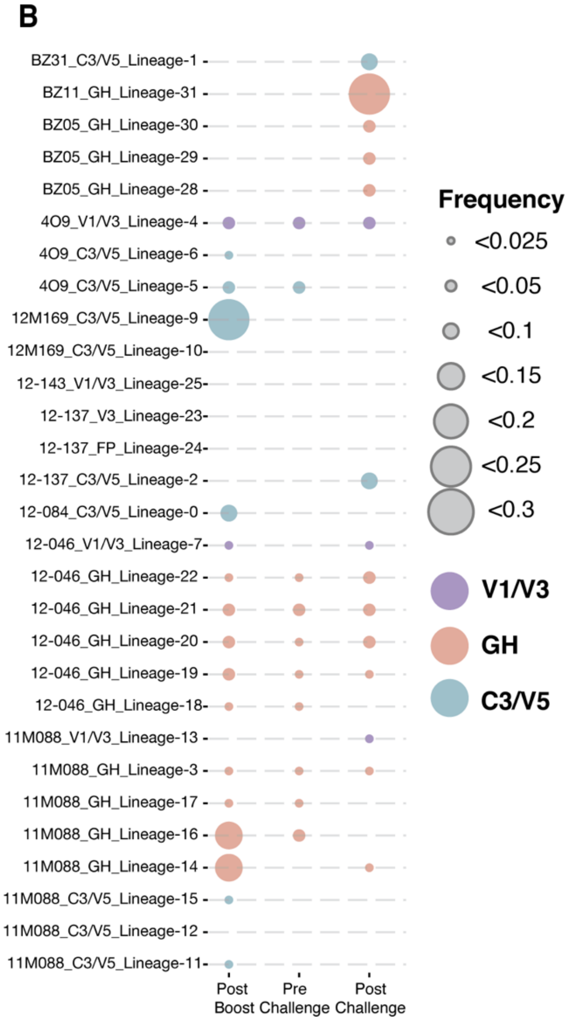

C

Replicate #1

	Post Boost	Pre-Challenge	Post Challenge
BZ31	N/A	N/A	465
12-084	0	N/A	N/A
12-137	0	0	26

Replicate #2

	Post Boost	Pre-Challenge	Post Challenge
BZ31	N/A	N/A	3056
12-084	176	N/A	N/A
12-137	0	0	55

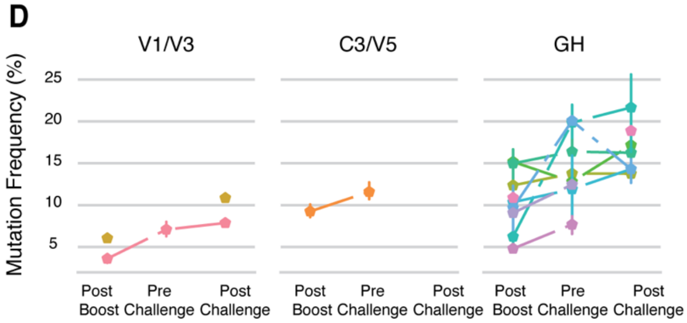

Figure S5. NAb frequency and mutation rate at multiple timepoints. Related to Figure 5. (**A**) Heavy chain and light chain amino acid sequence identities for rhesus mAbs. (**B**) nAb lineage heavy chain frequency (fractional abundance, defined as the number of unique antibody sequences in lineage divided by number of unique sequences in the repertoire at that timepoint) from corresponding animal NGS sequences at multiple timepoints: post-boost, pre-challenge, and post-challenge. (**C**) public C3/V5 heavy chain sequences found in animals BZ31, 12-084, 12-137 at multiple timepoints. Each replicate represents for an independent NGS run. N/A: not applicable. (**D**) Somatic hypermutation frequency of nAb lineages traced in (**B**) that can be found in more than one time-point. Each color represents for a separate lineage.

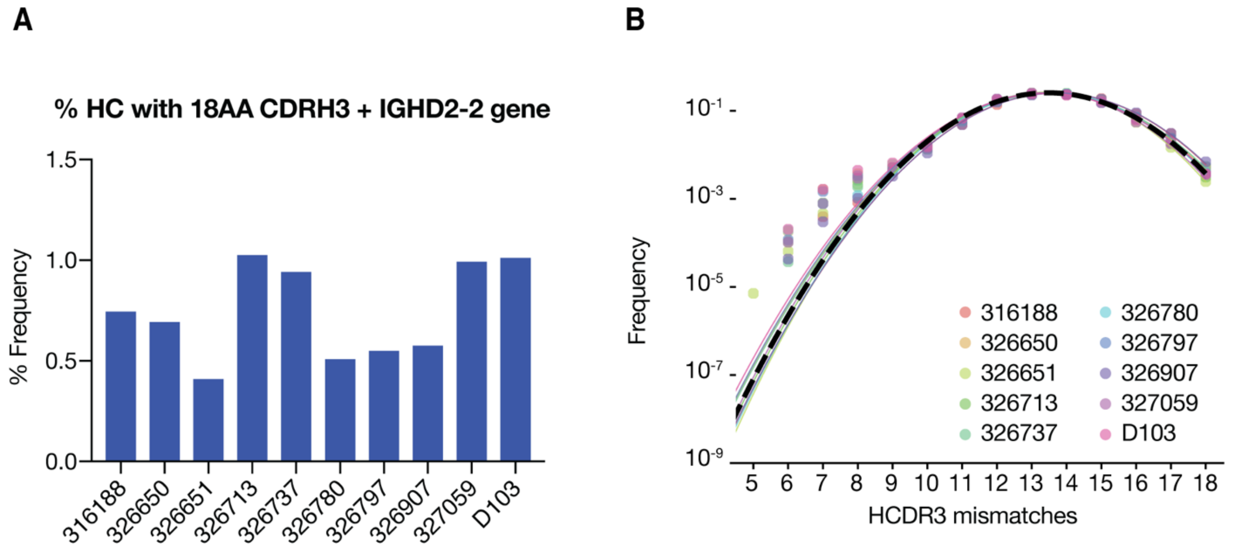

Figure S6. Frequency of similar public clonotype antibodies in the human B cell repertoire. Related to Figure 5. (**A**) Frequency of human heavy chain antibodies with an 18 amino acid CDRH3 length and containing the IGHD2-2 gene among ten healthy donors. (**B**) Distribution frequency of human heavy chain antibodies among 10 donors based on number of differences in amino acids compared to the most closely related NHP public clonotype antibody sequence.

Table S1. ELISA binding EC_{50} table of all rhesus mAbs against BG505 SOSIP, 398F1 SOSIP, and BG505 gp120. Related to Figure 1.

Group	Animal ID	mAb ID	Epitope	BG505 SOSIP EC_{50} (μg/mL)	BG505 gp120 EC_{50} (μg/mL)	398F1 SOSIP EC_{50} (μg/mL)
IMM	12-084	Rh084.1	C3/V5	0.62	0.22	>50
		Rh084.2	C3/V5	0.41	0.09	>50
		Rh084.3	C3/V5	0.66	0.17	>50
	4O9	Rh4O9.1	C3/V5	0.37	0.27	>50
		Rh4O9.2	C3/V5	0.16	0.03	>50
		Rh4O9.3	C3/V5	1.23	1.25	>50
		Rh4O9.4	C3/V5	0.39	0.03	>50
		Rh4O9.5	C3/V5	0.24	0.17	>50
	12-046	Rh046.1	GH	0.61	0.23	>50
		Rh046.2	GH	1.61	0.42	>50
		Rh046.3	GH	0.021	0.80	>50
	12-137	Rh137.1	C3/V5	0.60	0.20	>50
		Rh137.2	Interface	0.26	>50	>50
		Rh137.3	FP	0.08	>50	0.02
		Rh137.4	C3/V5	0.06	0.08	>50
		Rh137.5	C3/V5	0.02	0.03	>50
		Rh137.6	V3	5.32	0.46	0.22
	12-143	Rh143.1	V1/V3	54.64	24.30	>100
	12M169	Rh169.1	C3/V5	1.05	2.46	>50
	11M088	Rh088.1	GH	0.27	0.11	>50
NON-INF	4O9	Rh4O9.6	C3/V5	0.21	0.13	>50
		Rh4O9.7	V1/V3	0.41	0.76	>50
		Rh4O9.8	V1/V3	0.12	0.35	>50
	12-046	Rh046.4	GH	0.61	0.04	>50
		Rh046.5	GH	0.78	0.35	>50
		Rh046.6	V1/V3	0.24	0.11	>50
		Rh046.7	V1/V3	0.21	1.44	>50
INF	11M088	Rh088.2	GH	0.18	0.01	>50
		Rh088.3	GH	0.16	0.004	>50
		Rh088.4	GH	0.43	0.05	>50
		Rh088.5	V1/V3	0.23	0.33	>50
		Rh088.6	GH	0.36	0.02	>50
		Rh088.7	C3/V5	0.25	0.04	>50
		Rh088.8	C3/V5	0.35	0.08	>50
		Rh088.9	GH	0.51	0.09	>50
		Rh088.10	C3/V5	0.33	0.25	>50
		Rh088.11	GH	0.12	0.99	>50
	12M169	Rh129.2	C3/V5	0.74	0.11	>50
		Rh129.3	C3/V5	0.83	0.56	>50
		Rh129.4	C3/V5	0.08	0.05	>50
NI	BZ31	RhBZ31.1	C3/V5	0.20	0.60	>50
		RhBZ31.2	C3/V5	0.31	0.10	>50
		RhBZ31.3	C3/V5	0.95	0.45	>50
	BZ05	RhBZ05.1	GH	0.41	0.06	>50
		RhBZ05.2	GH	0.26	0.13	>50
		RhBZ05.3	GH	0.63	2.18	>50
	BZ11	RhBZ11.1	GH	0.08	0.60	>50

Table S2. Immunogenetic table of all rhesus mAbs. Related to Figure 1.

mAb ID	Epitope	Animal	Timepoint	VH gene	VD gene	VJ gene	CDRH3 aa	CDRH3 length	VH SHM % (nt)	VL/VK gene	VJ gene	CDRL3 aa	CDRL3 Length	VL/VK SHM %(nt)
Rh084.1	C3/V5	12-084	week 25	IGHV3-128*01	IGHD2-10*01	IGHJ5*01	TRDCSSSSCSWTYSRFDV	18	5	IGKV3-7*01	IGKJ3*01	YQYYSGYT	8	4
Rh084.2	C3/V5	12-084	week 25	IGHV3-128*01	IGHD2-10*01	IGHJ5*01	TRDCSSSACSSRYSRFDV	18	6	IGKV3-7*01	IGKJ3*01	YQYFSGYT	8	5
Rh084.3	C3/V5	12-084	week 25	IGHV3-128*01	IGHD2-10*01	IGHJ5*01	TRDCSSSSCSSRYSRFDV	18	6	IGKV3-7*01	IGKJ2*01	YQYFSGYT	8	4
Rh4O9.1	C3/V5	4O9	week 68	IGHV4-79*01	IGHD6-38*01	IGHJ4*01	ARAPTGS	7	6	IGKV1-25*01	IGKJ3*01	LQYFSLFT	8	7
Rh4O9.2	C3/V5	4O9	week 68	IGHV4-79*01	IGHD6-36*01	IGHJ4*01	ARAPTGN	7	10	IGKV1-25*01	IGKJ3*01	LQYFDLFT	8	7
Rh4O9.3	C3/V5	4O9	week 68	IGHV4-79*01	IGHD5-27*01	IGHJ4*01	ARGPTGN	7	9	IGKV1-25*01	IGKJ3*01	QQYFNFFS	8	7
Rh4O9.4	C3/V5	4O9	week 68	IGHV4-79*01	IGHD5-27*01	IGHJ4*01	VRGPTGN	7	9	IGKV1-25*01	IGKJ3*01	QQYFDLVT	8	7
Rh4O9.5	C3/V5	4O9	week 68	IGHV4-79*01	IGHD1-5*01	IGHJ4*01	ARSPTGN	7	9	IGKV1-25*01	IGKJ3*01	LQYFDLFT	8	5
Rh4O9.6	C3/V5	4O9	week 76	IGHV4-79*01	IGHD2-11*01	IGHJ4*01	ARTPTGD	7	9	IGKV1-25*01	IGKJ3*01	LQYFSLFT	8	6
Rh4O9.7	V1/V3	4O9	week 76	IGHV3-103*01	IGHD3-17*01	IGHJ4*01	ATTLGDYYVD	10	7	IGLV1-75*01	IGLJ3*01	AAWDDNLNGRV	11	4
Rh4O9.8	V1/V3	4O9	week 76	IGHV3-103*01	IGHD3-15*01	IGHJ4*01	TRTLGDYYLD	10	8	IGLV1-75*01	IGLJ3*01	AAWDDNLSGRI	11	4
Rh046.1	GH	12-046	week 26	IGHV4-149*01	IGHD4-24*01	IGHJ4*01	ARGGSSSLDF	10	10	IGKV1-50*01	IGKJ3*01	QQYNSDPFT	9	5
Rh046.2	GH	12-046	week 26	IGHV4-79*01	IGHD3-17*01	IGHJ4*01	AKGGEYAEY	9	11	IGKV1-50*01	IGKJ1*01	QQHKTYPWT	9	8
Rh046.3	GH	12-046	week 26	IGHV4-165*01	IGHD5-27*01	IGHJ4*01	ARGGQDFDY	9	13	IGKV1-50*01	IGKJ2*01	QQYNTYPYS	9	8
Rh046.4	GH	12-046	week 76	IGHV4-117*01	IGHD1-5*01	IGHJ4*01	ARGGSSSLDS	10	16	IGKV1-50*01	IGKJ3*01	HQYYRVPFT	9	7
Rh046.5	GH	12-046	week 76	IGHV4-149*01	IGHD1-8*01	IGHJ4*01	ARGGSEDFDS	10	12	IGLV1-55*01	IGLJ3*01	ASWDSRLNSVF	11	8
Rh046.6	V1/V3	12-046	week 76	IGHV3-127*01	IGHD2-11*01	IGHJ5*01	NTLPYCTGSRCYRGNRFDV	19	13	IGKV2-57*01	IGKJ1*01	MQGREFRT	8	9
Rh046.7	V1/V3	12-046	week 76	IGHV3-103*01	IGHD2-11*01	IGHJ5*01	NTLPYCTGGGCYRGNRFDV	19	11	IGKV2-57*01	IGKJ1*01	MNGLEFRT	8	9
Rh137.1	C3/V5	12-137	week 26	IGHV3-128*01	IGHD2-10*01	IGHJ5*01	TRDCSSSDCSSLFRRFDV	18	7	IGKV3-7*01	IGKJ2*01	YQYFSGYS	8	4
Rh137.2	interface	12-137	week 26	IGHV3-76*01	IGHD2-12*01	IGHJ4*01	AKDVPEYYSGGYPTFYFDF	19	11	IGKV1-49*01	IGKJ1*01	QQRNSHPPT	9	6
Rh137.3	FP	12-137	week 26	IGHV4-165*01	IGHD2-14*01	IGHJ4*01	ASGGYCSRGVCYAYFDS	17	8	IGKV1-50*01	IGKJ4*01	QQYNSAPLT	9	6
Rh137.4	C3/V5	12-137	week 76	IGHV3-128*01	IGHD2-10*01	IGHJ5*01	TRDCSSSDCSSSFRRFDV	18	7	IGKV3-7*01	IGKJ2*01	YQYFSGYT	8	7
Rh137.5	C3/V5	12-137	week 76	IGHV3-128*01	IGHD2-10*01	IGHJ5*01	TRDCSSSDCSSSFRRFDV	18	7	IGKV3-7*01	IGKJ2*01	YQYFSGYT	8	6
Rh137.6	V3	12-137	week 26	IGHV3-103*01	IGHD6-33*01	IGHJ5*02	SRAGRWYSSAWSSLDV	16	9	IGKV1-44*01	IGKJ3*01	LQGYSTPFT	9	7
Rh143.1	V1/V3	12-143	week 26	IGHV4-174*01	IGHD3-15*01	IGHJ4*01	TRDQDNYAYYYYFDC	15	2	IGKV1-25*01	IGKJ4*01	QQYNSLPLT	9	4
Rh169.1	C3/V5	12M169	week 28	IGHV3-28*01	IGHD6-39*01	IGHJ4*01	AVIAAATFVDY	11	5	IGKV1-51*01	IGKJ1*01	LQGLFTPWT	9	3
Rh169.2	C3/V5	12M169	week 86	IGHV1-58*01	IGHD3-15*01	IGHJ4*01	TRGVRHDYAYYYTNVDF	17	11	IGLV2-29*01	IGLJ1*01	SSFAGSHTFI	10	9
Rh169.3	C3/V5	12M169	week 86	IGHV3-28*01	IGHD6-39*01	IGHJ4*01	AAIAAATFIDY	11	11	IGKV1-44*01	IGKJ1*01	LQGFFTPWT	9	5
Rh169.4	C3/V5	12M169	week 86	IGHV1-58*01	IGHD3-15*01	IGHJ4*01	ARGVRHDYAEYYTTFDS	17	14	IGLV2-29*01	IGLJ1*01	SSYAGSNTFI	10	9
Rh088.1	GH	11M088	week 28	IGHV4-79*01	IGHD3-15*01	IGHJ4*01	STRMGET	7	11	IGKV3-11*01	IGKJ2*01	QQGSNWPYS	9	7
Rh088.2	GH	11M088	week 86	IGHV4-149*01	IGHD6-33*01	IGHJ3*01	ARKQDNFDF	9	11	IGKV1-50*01	IGKJ1*01	QQYYNEPWT	9	5
Rh088.3	GH	11M088	week 86	IGHV4-149*01	IGHD1-2*01	IGHJ3*01	ARKEDDFDF	9	12	IGKV1-50*01	IGKJ1*01	QQYYNDPWT	9	6
Rh088.4	GH	11M088	week 86	IGHV4-149*01	IGHD1-2*01	IGHJ3*01	ARKEDDFDF	9	11	IGKV1-50*01	IGKJ1*01	QQYNNDPWT	9	5
Rh088.5	V1/V3	11M088	week 86	IGHV3-172*01	IGHD2-10*01	IGHJ4*01	TTGEYFLHLDY	11	16	IGKV1-21*01	IGKJ3*01	LQHHTYPFT	9	12
Rh088.6	GH	11M088	week 86	IGHV4-149*01	IGHD1-2*01	IGHJ3*01	ARKEDDFDF	9	10	IGKV1-50*01	IGKJ1*01	QQYNNDPWT	9	5
Rh088.7	C3/V5	11M088	week 86	IGHV1-58*01	IGHD3-17*01	IGHJ3*01	ARGAVRGAYYYVTFDL	16	15	IGLV2-18*01	IGLJ1*01	SSYAGSGTFI	10	7
Rh088.8	C3/V5	11M088	week 86	IGHV1-58*01	IGHD1-4*01	IGHJ6*01	ARVVRTGLEQLLAGALDS	18	11	IGKV3-52*01	IGKJ2*01	QKYNDWPYS	9	5
Rh088.9	GH	11M088	week 86	IGHV4-149*01	IGHD6-33*01	IGHJ3*01	ARKQEDFDF	9	9	IGKV1-50*01	IGKJ1*01	QQYDNDPWT	9	8
Rh088.10	C3/V5	11M088	week 86	IGHV1-58*01	IGHD3-15*01	IGHJ4*01	ARATRDDNTYYYTHFDT	17	11	IGLV2-10*01	IGLJ1*01	CSYEASDTFI	10	6
Rh088.11	GH	11M088	week 86	IGHV3-50*01	IGHD2-11*01	IGHJ4*01	VRDQMEVVVRNSEIDY	16	7	IGKV3-9*01	IGKJ3*01	HQGNITPFT	9	7
RhBZ31.1	C3/V5	BZ31	week 86	IGHV3-128*01	IGHD2-10*01	IGHJ5*01	SRDCSSTDCSSSYSRFDV	18	3	IGKV3-7*01	IGKJ1*01	FQYFSGWT	8	3
RhBZ31.2	C3/V5	BZ31	week 86	IGHV3-128*01	IGHD2-10*01	IGHJ5*01	TRDCSSDFCSSSYNRFNV	18	4	IGKV3-7*01	IGKJ1*01	FQYYSGWT	8	3
RhBZ31.3	C3/V5	BZ31	week 86	IGHV3-128*01	IGHD2-10*01	IGHJ5*01	TRDCSSTSCSSSYSRFDV	18	4	IGKV3-7*01	IGKJ1*01	FQYFSGWT	8	4
RhBZ05.1	GH	BZ05	week 86	IGHV4-79*01	IGHD6-38*01	IGHJ4*01	VRDKGWD	7	12	IGKV3-11*01	IGKJ1*01	QQESNWPWT	9	6
RhBZ05.2	GH	BZ05	week 86	IGHV4-79*01	IGHD6-38*01	IGHJ4*01	ARDKGWD	7	9	IGKV3-11*01	IGKJ1*01	QQESNWPWT	9	8
RhBZ05.3	GH	BZ05	week 86	IGHV4-61*01	IGHD3-41*01	IGHJ1*01	ARDLDGGYNFWSGPPGYFEF	20	6	IGKV1-21*01	IGKJ2*01	LQHNTYPYS	9	6
RhBZ11.1	GH	BZ11	week 86	IGHV4-150*01	IGHD5-28*01	IGHJ4*01	AKERTTVQFDY	11	9	IGLV2-12*01	IGLJ1*01	SSYSSDDIYI	10	6

Table S3. Neutralization IC_{50} table of all rhesus mAbs against WT BG505 and MG505.A2 viruses, and variants with substitutions at the 241/289 GH, the C3/V5 epitope, the V1/V3 epitope, and the fusion peptide epitope, respectively. Related to Figure 2.

Epitope	Group	mAb ID	Maximum Neut (%)	WT IC50 (ug/mL)		241/289 GH		C3/V5			V1/V3		FP
				BG505	MG505.A2	BG505 N289	BG505 N241N289	BG505 TI357KT	MG505 KTII357TIIR	BG505 T465N	BG505 CTNN	BG505 Loop H3	BG505 N611A
C3/V5	IMM	Rh084.1	100	0.03	>50	0.25	0.56	>50	>50	0.03	0.22	0.35	0.09
	IMM	Rh084.2	100	0.09	>50	0.20	0.15	>50	7.88	0.30	0.07	0.11	0.02
	IMM	Rh084.3	100	0.10	>50	0.27	0.23	>50	1.94	0.32	0.40	0.10	0.02
	IMM	Rh4O9.1	100	0.07	>50	0.11	0.12	>50	1.57	0.27	0.10	0.04	0.09
	IMM	Rh4O9.2	100	0.05	>50	0.04	0.10	>50	1.11	0.03	0.04	0.04	0.07
	IMM	Rh4O9.3	100	0.15	>50	0.14	0.27	>50	>50	0.15	0.16	0.20	0.07
	IMM	Rh4O9.4	100	0.04	>50	0.05	0.03	>50	1.53	0.23	0.02	0.02	0.02
	IMM	Rh4O9.5	100	0.03	>50	0.08	0.26	>50	2.28	0.08	0.22	0.22	0.06
	NON-INF	Rh4O9.6	100	0.07	>50	0.12	0.05	3.97	1.80	0.35	0.04	0.06	0.09
	IMM	Rh137.1	100	0.08	>50	0.10	0.30	>50	14.21	5.10	0.18	0.10	0.13
	IMM	Rh137.4	100	0.02	>50	0.12	0.05	>50	1.69	0.17	0.03	0.04	0.08
	IMM	Rh137.5	100	0.91	>50	0.64	0.25	>50	6.54	3.39	0.19	0.25	0.38
	IMM	Rh169.1	100	0.28	>50	0.46	0.97	13.57	27.46	>50	1.43	0.55	0.44
	INF	Rh088.7	95	0.04	>50	0.02	0.08	>50	2.40	>50	0.05	0.11	0.04
	INF	Rh088.8	98	0.09	>50	0.07	0.13	>50	10.11	14.30	0.11	0.36	0.13
	INF	Rh088.10	95	0.08	>50	0.20	0.33	>50	15.51	>50	0.14	0.26	0.13
	INF	Rh129.2	98	0.06	>50	0.12	0.04	2.19	0.95	28.89	0.05	0.08	0.12
	INF	Rh129.3	100	0.10	>50	0.16	0.12	0.70	1.40	>50	0.06	0.10	0.16
	INF	Rh129.4	99	0.06	>50	0.64	0.11	>50	1.59	>50	0.04	0.06	0.10
	NI	RhBZ31.1	100	0.04	>50	0.05	0.27	>50	1.26	1.47	0.12	0.07	0.26
	NI	RhBZ31.2	100	0.02	>50	0.02	0.16	>50	0.79	0.42	0.09	0.14	0.04
	NI	RhBZ31.3	100	0.06	>50	0.32	3.91	>50	2.81	0.69	3.21	4.78	0.08
GH	IMM	Rh046.1	60	5.44	>50	>50	>50	>50	>50	12.97	37.22	>50	19.26
	IMM	Rh046.2	53	9.89	>50	>50	>50	>50	>50	29.38	>50	>50	42.05
	IMM	Rh046.3	50	14.36	>50	>50	>50	>50	>50	39.21	31.19	32.15	>50
	NON-INF	Rh046.4	51	19.96	>50	>50	>50	>50	>50	3.36	22.87	>50	32.18
	NON-INF	Rh046.5	50	17.54	>50	>50	>50	9.82	>50	31.13	28.75	35.45	19.51
	IMM	Rh088.1	54	11.03	>50	>50	>50	>50	>50	20.69	45.41	39.32	>50
	INF	Rh088.2	62	5.36	>50	>50	>50	24.45	>50	41.66	>50	27.76	1.64
	INF	Rh088.3	50	12.56	>50	>50	>50	>50	>50	>50	23.35	8.94	10.55
	INF	Rh088.4	53	8.69	>50	>50	>50	>50	>50	>50	25.13	2.52	24.09
	INF	Rh088.6	50	11.24	>50	>50	>50	>50	>50	7.46	5.25	4.28	14.73
	INF	Rh088.9	51	3.24	>50	>50	>50	10.47	>50	>50	44.36	45.51	2.07
	INF	Rh088.11	85	0.46	>50	18.42	>50	0.43	>50	4.29	1.50	0.97	1.09
	NI	RhBZ05.1	63	2.32	>50	>50	>50	32.30	>50	47.71	15.73	>50	33.41
	NI	RhBZ05.2	57	9.36	>50	>50	>50	42.68	>50	36.94	>50	>50	>50
	NI	RhBZ05.3	53	12.21	>50	16.01	>50	>50	>50	>50	24.81	>50	>50
	NI	RhBZ11.1	75	0.74	>50	0.24	>50	0.27	>50	15.07	27.75	20.05	1.33
	IMM	Rh143.1	70	8.42	36.78	46.35	>100	44.54	>100	36.53	>100	>100	38.22
V1/V3	NON-INF	Rh4O9.7	100	0.08	0.24	0.13	0.11	0.28	0.49	0.05	>50	>50	0.38
	NON-INF	Rh4O9.8	100	0.12	0.19	0.17	0.10	0.29	0.17	0.12	>50	>50	0.15
	NON-INF	Rh046.6	100	0.02	0.06	0.08	0.01	0.06	0.10	0.07	>50	>50	0.04
	NON-INF	Rh046.7	100	0.09	0.40	0.50	0.06	0.29	0.64	0.45	>50	>50	0.10
	INF	Rh088.5	100	0.04	0.10	0.03	0.03	0.09	0.14	0.12	>50	>50	0.06
Interface/FP	IMM	Rh137.2	95	3.04	2.98	5.76	23.95	2.10	2.33	4.37	38.51	32.79	0.17
	IMM	Rh137.3	34	>50	>50	>50	>50	>50	>50	>50	>50	>50	1.27
V3	IMM	Rh137.6	0	>50	>50	N/A	N/A	N/A	N/A	N/A	N/A	N/A	>50

N/A: not applicable

Table S4. Neutralization IC_{50} (µg/mL) breadth of all rhesus mAbs including PGT121 as a control against a global panel of HIV isolates from multiple clades. Related to Figure 2.

	398F1	246F3	CNE55	CNE8	X2278	TRO11	BJOX2000	CH119	CE0217	CE1176	25710	X1632
Clade	A	AC	AE	AE	B	B	BC	BC	C	C	C	G
Rh084.1	>50	>50	>50	>50	>50	>50	>50	>50	>50	>50	>50	>50
Rh084.2	>50	>50	>50	>50	>50	>50	>50	>50	>50	>50	>50	>50
Rh084.3	>50	>50	>50	>50	>50	>50	>50	>50	>50	>50	>50	>50
Rh4O9.1	>50	>50	>50	>50	>50	>50	>50	>50	>50	>50	>50	>50
Rh4O9.2	>50	>50	>50	>50	>50	>50	>50	>50	>50	>50	>50	>50
Rh4O9.3	>50	>50	>50	>50	>50	>50	>50	>50	>50	>50	>50	>50
Rh4O9.4	>50	>50	>50	>50	>50	>50	>50	>50	>50	>50	>50	>50
Rh4O9.5	>50	>50	>50	>50	>50	>50	>50	>50	>50	>50	>50	>50
Rh046.1	>50	>50	>50	>50	>50	>50	>50	>50	>50	>50	>50	>50
Rh046.2	>50	>50	>50	>50	>50	>50	>50	>50	>50	>50	>50	>50
Rh046.3	>50	>50	>50	>50	>50	>50	>50	>50	>50	>50	>50	>50
Rh137.1	>50	>50	>50	>50	>50	>50	>50	>50	>50	>50	>50	>50
Rh137.2	>50	>50	>50	>50	>50	>50	>50	>50	>50	>50	>50	>50
Rh137.3	>50	>50	>50	>50	>50	>50	>50	>50	>50	>50	>50	>50
Rh137.4	>50	>50	>50	>50	>50	>50	>50	>50	>50	>50	>50	>50
Rh137.5	>50	>50	>50	>50	>50	>50	>50	>50	>50	>50	>50	>50
Rh137.6	18.00	>50	>50	>50	>50	>50	>50	>50	>50	>50	>50	>50
Rh143.1	>50	>50	>50	>50	>50	>50	>50	>50	>50	>50	>50	>50
Rh169.1	>50	>50	>50	>50	>50	>50	>50	>50	>50	>50	>50	>50
Rh088.1	>50	>50	>50	>50	>50	>50	>50	>50	>50	>50	>50	>50
Rh4O9.6	>50	>50	>50	>50	>50	>50	>50	>50	>50	>50	>50	>50
Rh4O9.7	>50	>50	>50	>50	>50	>50	>50	>50	>50	>50	>50	>50
Rh4O9.8	>50	>50	>50	>50	>50	>50	>50	>50	>50	>50	>50	>50
Rh046.4	>50	>50	>50	>50	>50	>50	>50	>50	>50	>50	>50	>50
Rh046.5	>50	>50	>50	>50	>50	>50	>50	>50	>50	>50	>50	>50
Rh046.6	>50	>50	>50	>50	>50	>50	>50	>50	>50	>50	>50	>50
Rh046.7	>50	>50	>50	>50	>50	>50	>50	>50	>50	>50	>50	>50
Rh088.2	>50	>50	>50	>50	>50	>50	>50	>50	>50	>50	>50	>50
Rh088.3	>50	>50	>50	>50	>50	>50	>50	>50	>50	>50	>50	>50
Rh088.4	>50	>50	>50	>50	>50	>50	>50	>50	>50	>50	>50	>50
Rh088.5	>50	>50	>50	>50	>50	>50	>50	>50	>50	>50	>50	>50
Rh088.6	>50	>50	>50	>50	>50	>50	>50	>50	>50	>50	>50	>50
Rh088.7	>50	>50	>50	>50	>50	>50	>50	>50	>50	>50	>50	>50
Rh088.8	>50	>50	>50	>50	>50	>50	>50	>50	>50	>50	>50	>50
Rh088.9	>50	>50	>50	>50	>50	>50	>50	>50	>50	>50	>50	>50
Rh088.10	>50	>50	>50	>50	>50	>50	>50	>50	>50	>50	>50	>50
Rh088.11	>50	>50	>50	>50	>50	>50	>50	>50	>50	>50	>50	>50
Rh169.2	>50	>50	>50	>50	>50	>50	>50	>50	>50	>50	>50	>50
Rh169.3	>50	>50	>50	>50	>50	>50	>50	>50	>50	>50	>50	>50
Rh169.4	>50	>50	>50	>50	>50	>50	>50	>50	>50	>50	>50	>50
RhBZ31.1	>50	>50	>50	>50	>50	>50	>50	>50	>50	>50	>50	>50
RhBZ31.2	>50	>50	>50	>50	>50	>50	>50	>50	>50	>50	>50	>50
RhBZ31.3	>50	>50	>50	>50	>50	>50	>50	>50	>50	>50	>50	>50
RhBZ05.1	>50	>50	>50	>50	>50	>50	>50	>50	>50	>50	>50	>50
RhBZ05.2	>50	>50	>50	>50	>50	>50	>50	>50	>50	>50	>50	>50
RhBZ05.3	>50	>50	>50	>50	>50	>50	>50	>50	>50	>50	>50	>50
RhBZ11.1	>50	>50	>50	>50	>50	>50	>50	>50	>50	>50	>50	>50
PGT121	0.003	>50	>50	>50	0.02	0.001	0.07	0.02	0.01	0.02	0.01	>50
